# Creatinine accelerates APAP-induced liver damage by increasing oxidative stress through ROS/JNK signaling pathway

**DOI:** 10.3389/fphar.2022.959497

**Published:** 2022-08-24

**Authors:** Fang Liu, Yan Liu, Qifeng Peng, Guodong Wang, Qing Tan, Zhongyue Ou, Qishan Xu, Chixiang Liu, Daming Zuo, Jianbo Zhao

**Affiliations:** ^1^ Division of Vascular and Interventional Radiology, Department of General Surgery, Nanfang Hospital, Southern Medical University, Guangzhou, Guangdong, China; ^2^ Department of Medical Laboratory, School of Laboratory Medicine and Biotechnology, Southern Medical University, Guangzhou, Guangdong, China; ^3^ Syndrome Laboratory of Integrated Chinese and Western Medicine, School of Chinese Medicine, Southern Medical University, Guangzhou, Guangdong, China; ^4^ Department of Oncology, Liuzhou Workers Hospital, Liuzhou, China; ^5^ Department of Blood Transfusion, Nanfang Hospital, Southern Medical University, Guangzhou, Guangdong, China; ^6^ Department of Immunology, School of Basic Medical Sciences, Southern Medical University, Guangzhou, Guangdong, China

**Keywords:** creatinine, acetaminophen, reactive oxygen species, C-jun N-terminal kinase, hepatotoxicity

## Abstract

Serum creatinine is an endogenous biomarker to estimate glomerular filtration rate (GFR) and is commonly used to assess renal function in clinical practice. Acetaminophen (APAP), the most available analgesic and antipyretic medication, is recommended as the drug of choice for pain control in patients with renal diseases. However, an overdose of APAP can lead to severe acute liver injury, which is also the most common cause of acute liver failure in western countries. The role of creatinine in APAP-induced liver injury is unclear and should be further explored. Herein, clinical data on patients with drug-induced liver injury revealed that the creatinine concentration between 82-442 μmol/L for female and 98–442 μmol/L for male is positively correlated with alanine aminotransferase (ALT), aspartate aminotransferase (AST). While there was no correlation between creatinine and ALT and AST when creatinine concentration is over 442 μmol/L. In addition, mice were administrated with creatinine intraperitoneally for 1 week before APAP injection to investigated the pathophysiological role of creatinine in APAP-induced acute liver injury. The results showed that creatinine administration aggravated hepatic necrosis and elevated serum lactate dehydrogenase (LDH) and ALT levels in mice upon APAP injection. The mechanism study demonstrated that creatinine could increase the production of reactive oxygen activation (ROS) and the activation of c-Jun N-terminal kinase (JNK). Furthermore, the liver injury was alleviated and the difference between APAP-treated mice and APAP combined with creatinine-treated mice was blunted after using specific ROS and JNK inhibitors. Significantly, creatinine stimulation aggravates APAP-induced cell death in HepaRG cells with the same mechanism. In summary, this study proposed that creatinine is closely related with liver function of drug-induced liver injury and exacerbates APAP-induced hepatocyte death by promoting ROS production and JNK activation, thus providing new insight into the usage of APAP in patients with kidney problems.

## Introduction

Acetaminophen (APAP) is commonly used as an antipyretic and analgesic in clinical around the world (Larson et al., 2005; Jaeschke, 2015). At therapeutic dose, APAP can be glucuronidated or sulfated in the liver or neutralized by conjugation with glutathione (GSH) through metabolism into the electrophilic intermediate N-acetyl-p-benzoquinoneimine (NAPQI) and then excreted in the urine. However, exposure to an overdose of APAP can lead to an accumulation of NAPQI and a depletion of GSH. Subsequently, excess NAPQI binds directly to cell proteins to produce NAPQI-protein complexes, leading to mitochondrial aberration, oxidative stress, and nuclear DNA damage, which drives hepatocyte damage and necrosis (Jaeschke, 1990; Nelson, 1990; Botta et al., 2006). Indeed, the mitochondrial ROS generation resulting from hepatic GSH depletion in response to APAP can activate the C-jun-N-terminal kinase (JNK) pathway (Cover et al., 2005; Theruvath et al., 2008; Singh et al., 2009; Du et al., 2016). Additionally, JNK activation in APAP toxicity leads to the release and translocation of Bax to mitochondria (Wang et al., 2018; Li et al., 2019).

Serum creatinine is a product of muscle catabolism and is mainly secreted by tubules, which universally used to be evidence of any health issue related with renal, muscular function (Perrone et al., 1992; Wyss and Kaddurah-Daouk, 2000). It was revealed that the high level of serum creatinine is relative to several diseases, including chronic kidney disease (CKD), different types of muscular disorders, cardiovascular problems (Cánovas et al., 2019). Emerging evidence suggested serum creatinine is also closely associated with the progression of liver diseases. A cross-sectional analysis in a middle-aged and older Chinese population showed that subjects with non-alcoholic fatty liver disease (NAFLD) had higher serum creatinine than those without NAFLD. Moreover, serum creatinine levels were correlated with the levels of ALT and AST (Niu et al., 2021). The creatinine level in serum is also applied to define hepatorenal syndrome (HRS) in patients with progressive liver disease (Boyer et al., 2011). Interestingly, a previous report showed that patients with severe hepatic failure have abnormally low serum creatinine concentrations (Takabatake et al., 1988). It is worthy to mention that APAP is the drug often recommended for occasional use in patients with kidney diseases. However, the potential function of serum creatinine in APAP-associated acute liver injury is undefined, which remains further investigation. In the present study, the direct connection between creatinine and liver function of drug-induced liver injury and the underlying mechanism of creatinine in APAP-induced liver injury were studied.

## Methods and materials

### Clinical data

**FIGURE 1:**
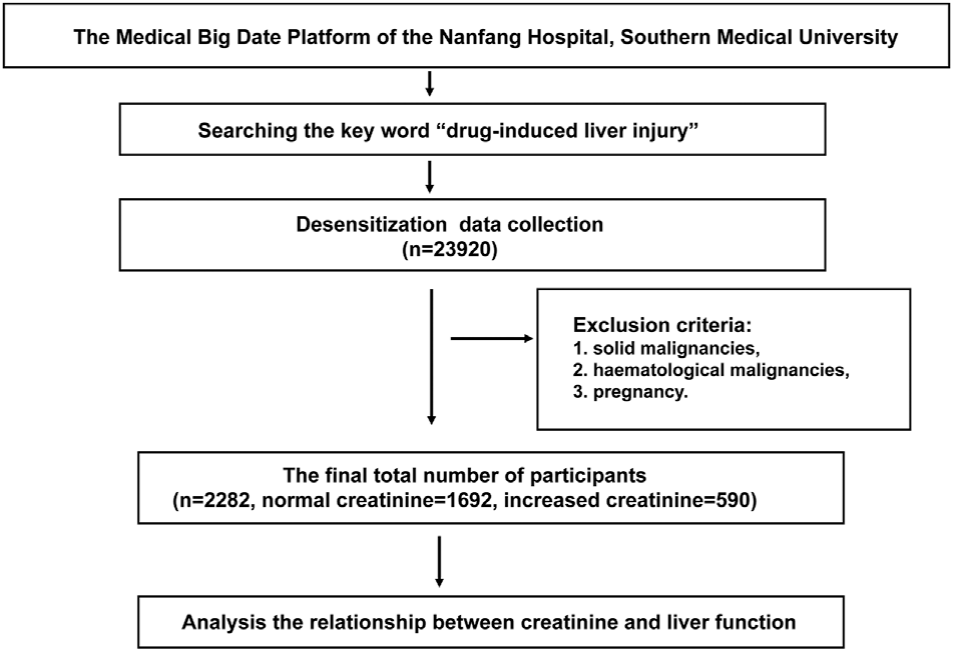
Flow chart of the clinical study. Clinical data of 23,920 patients who have been diagnosed as drug-induced liver injury in the Medical Big Date Platform of the Nanfang Hospital of Southern Medical University were collected. After excluding patients with recurrent and metastatic tumors, malignancies, pregnancy, 2,282 patients (1,692 with normal creatinine and 590 with increased creatinine) were identified eligible in this study (Figure 1).

Clinical information of 23,920 patients according to international classification of diseases code-10 (ICD-10 code K71.901) who had received a drug-induced liver injury diagnosis were obtained from the clinical information system in Nanfang Hospital, Southern Medical University. The diagnosis criteria for drug-induced liver injury refers to the “Guidelines for the management of drug-induced liver injury” from Chinese Medical Association. Patients complicated with the following diseases were excluded: 1) solid or haematological malignancies, 2) pregnancy. Then 2,282 patients (1,692 with normal creatinine and 590 with increased creatinine) were identified eligible in this study (Figure 1). These patients were separated into six groups according to CKD staging guidelines based on serum creatinine, including group 1: 41–81 μmol/L for female (*n* = 657) and 57–97 μmol/L for male (*n* = 1,035). Group 2: 82–132 μmol/L for female (*n* = 90) and 98–132 μmol/L for male (*n* = 220), group 3: 133–176 μmol/L for female (*n* = 28) and male (*n* = 91), group 4: 177–442 μmol/L for female (*n* = 18) and male (n = 61), group 5: 443–707 μmol/L for female (*n* = 28) and male (*n* = 22), group 6: >707 μmol/L for female (*n* = 10) and male (*n* = 22). A detailed description of the patients is provided in the Supplementary Material (Table 1). In addition, the employed data belong to in-hospital desensitization text data, not involving any patient privacy information, and only for scientific research.

**TABLE 1:** Main baseline clinical data.

Variables	Female	Male
Age (years)	40.96 ± 16.38	41.76 ± 16.54
gender (%)	36% (831/2,282)	64% (1,451/2,282)
Creatinine (μmol/L)	96.10 ± 133.41	113.90 ± 132.99
AST (U/L)	200.63 ± 264.22	182.17 ± 225.95
ALT (U/L)	228.15 ± 160.05	272.77 ± 253.29
TBIL (μmol/L)	80.92 ± 125.49	101.02 ± 159.33
ALB (g/L)	34.34 ± 5.99	34.72 ± 6.40
ALP (mmol/L)	185.22 ± 186.41	163.67 ± 107.63

### Bioinformatic analysis

Two available human datasets (GSE30718 and GSE62792) were retrieved using the Gene Expression Omnibus (GEO) database https://www.ncbi.nlm.nih.gov/geo and the Array Express database www.ebi.ac.uk/arrayexpress. The GSE30718 dataset contained 28 patients with acute kidney injury and 3 healthy people. The GSE62792 dataset contained 12 patients with CKD and 6 healthy people. Gene expression profiles of high serum creatinine patients (acute kidney injury or CKD) and normal people (healthy people) were obtained and compared to calculate the enrichment score and *p* value of each KEGG pathway about oxidative stress by a non-parametric Wilcoxon rank sum test.

#### Animals

Male C57BL/6 mice were purchased from the Animal Institute of Nanfang Hospital, Southern Medical University of Guangdong Province and kept under specific pathogen-free conditions. The animal experiments were approved by the Welfare and Ethical Committee for Experimental Animal Care of Nanfang Hospital of Southern Medical University. All experiments were carried out under the approved protocol (NFYY-2021-0342).

#### Mice model

Mice (male, 8–10 weeks old) were randomized into 8 groups (*n* = 3/group): Control group, Creatinine group, APAP group, APAP + Creatinine group, APAP + N-acetyl-L-cysteine (NAC) group, APAP + Creatinine + NAC group, APAP + SP600125 group and APAP + Creatinine + SP600125 group. For the creatinine pre-treatment groups, the mice were injected intraperitoneally with creatinine (C4255, Sigma-Aldrich, United States ) at the dose of 16 mg/g for 1 week before APAP treatment. For the APAP-induced acute liver injury model, mice were fasted overnight and then administered with APAP (A7085, Sigma-Aldrich, United States ) intraperitoneally at the dose of 400 mg/kg. The other groups were intraperitoneally administrated with equal volume of 0.9% saline solution. In some experiments, mice were given 150 mg/kg of NAC (A7250, Sigma-Aldrich, United States ) 1 h before the APAP administration to minimize the effect of ROS. In some cases, the pharmacologic inhibitor SP600125 (S1460, Selleck, United States ) was used at a dose of 15 mg/kg 1 h prior to the APAP injection to inhibit the activation of JNK. Mice were finally intraperitoneal injected with 1% pentobarbital (50 mg/kg) for anaesthetization and sacrificed at 6 h for flow cytometric assay and 24 h for biochemical and histological detection.

#### Reagents and antibodies

Colorimetric TUNEL assay kit (C1098, Beyotime, China) and Mitochondrial membrane potential assay kit (M36008, Invitrogen, United States ) as well as JC-1 (C2006, Beyotime, China) were purchased. Annexin V-FITC/PI apoptosis kits got from MultiSciences (AP101, Hangzhou, China). The primary antibodies against Bcl2(66,799), Bax (50,599) and β-actin (66,009) were obtained from Proteintech (Wuhan, China). The primary antibodies, including the antibodies against phospho-JNK (81E11) and JNK (9,252) were purchased from Cell Signaling Technology (Danvers, MA, United States ). The kits including ALT (C009-2-1), lactate dehydrogenase (LDH, A020-02-2), Malondialdehyde (MDA, A003-1-2) and GSH (A0062-1) were purchased from Jiancheng Biotech (Nanjing, China). Cell-Counting-Kit-8 (K1018) was bought from APE-BIO.

#### Cell culture and treatment

HepaRG cells were purchased from Biopredic International (Rennes, France) and cultured with RPMI 1640 medium containing 10% fetal bovine serum (FBS). The cells were stimulated with 20 mM APAP in the presence or absence of 20 mM creatinine. For cell cytotoxicity experiment, cells were seeded in 96-well culture plates and then stimulated with different concentration of creatinine to assess cell viability using CCK8 at 24, 48 h.

#### Histological assessment

Paraffin-embedded liver tissues were stained with hematoxylin and eosin (H&E) to assay the severity of the liver injury. The TdT-mediated dUTP Nick-End Labeling method (TUNEL) was used to evaluate the apoptosis of hepatocytes in APAP-induced liver injury. The experiments were performed according to the manufacturer’s instructions.

#### ALT and lactate dehydrogenase detection

Mice sera were collected at 24 h after the APAP challenge with or without creatinine pretreatment. The activities of serum ALT and LDH were assayed with the commercial kits, referring to the manufacturer’s instruction.

#### Malondialdehyde and generation resulting from hepatic Assay

Aliquot liver tissues were homogenized with ultrasound in ice-cold phosphate-buffered saline (PBS). The MDA and GSH levels in liver homogenates were detected using commercial kits, referring to the manufacturer’s instruction.

#### Western blotting analysis

Protein samples were obtained from mice live tissues or HepaRG cells. The equal amounts of protein were separated on 10% sodium dodecyl sulfate-polyacrylamide gel electrophoresis (SDS-PAGE) and then transferred onto PVDF (Millipore, Germany) membranes. Then the PVDF membranes were incubated overnight at 4°C with primary antibodies after blocking with bovine serum albumin (BSA, 5%) for 1 h at room temperature. Subsequently, the membranes were incubated with the horseradish peroxidase-conjugated corresponding secondary antibody for 1 h at room temperature and detected the target protein expression with enhanced chemiluminescence (Thermo Fisher, United States ).

#### Flow cytometric assay

Hepatocytes after the APAP challenge were isolated using the two-step pronase-collagenase perfusion method. The HepaRG cells after exposure to APAP with or without creatinine for 6 h were collected. The intracellular ROS and Mito-sox, as well as the mitochondrial membrane potential and cellular apoptosis were evaluated using commercial kits, referring to the manufacturer’s instruction.

#### Fluorescence Monitoring

The HepaRG treated with APAP in presence or absence of creatinine were collected and incubated with 10 μg/ml of the JC-1 dye at 37°C for 20 min. The mitochondrial membrane potential in cells was analyzed using fluorescence microscopy (Olympus, Shinjuku, Tokyo, Japan).

#### Statistical analysis

The clinical data were expressed as the mean plus or minus the standard deviation (SD) and compared using non-parametric t-tests. The Spearman correlation coefficient was used to analyse the relationships between creatinine and ALT and AST. The experimental data were expressed as mean ± SEM. For the comparison between multiple groups, the One-way ANOVA was used. Differences between two groups in the experiments were evaluated by Student’s t-test. Statistical analysis was performed using GraphPad Prism (GraphPad Software, La Jolla, CA,United States), and differences were considered statistically significant at *p* < 0.05.

## Results

### Serum creatinine concentration is correlated with ALT and AST in patients with diagnosis of drug-induced liver injury

**FIGURE 2:**
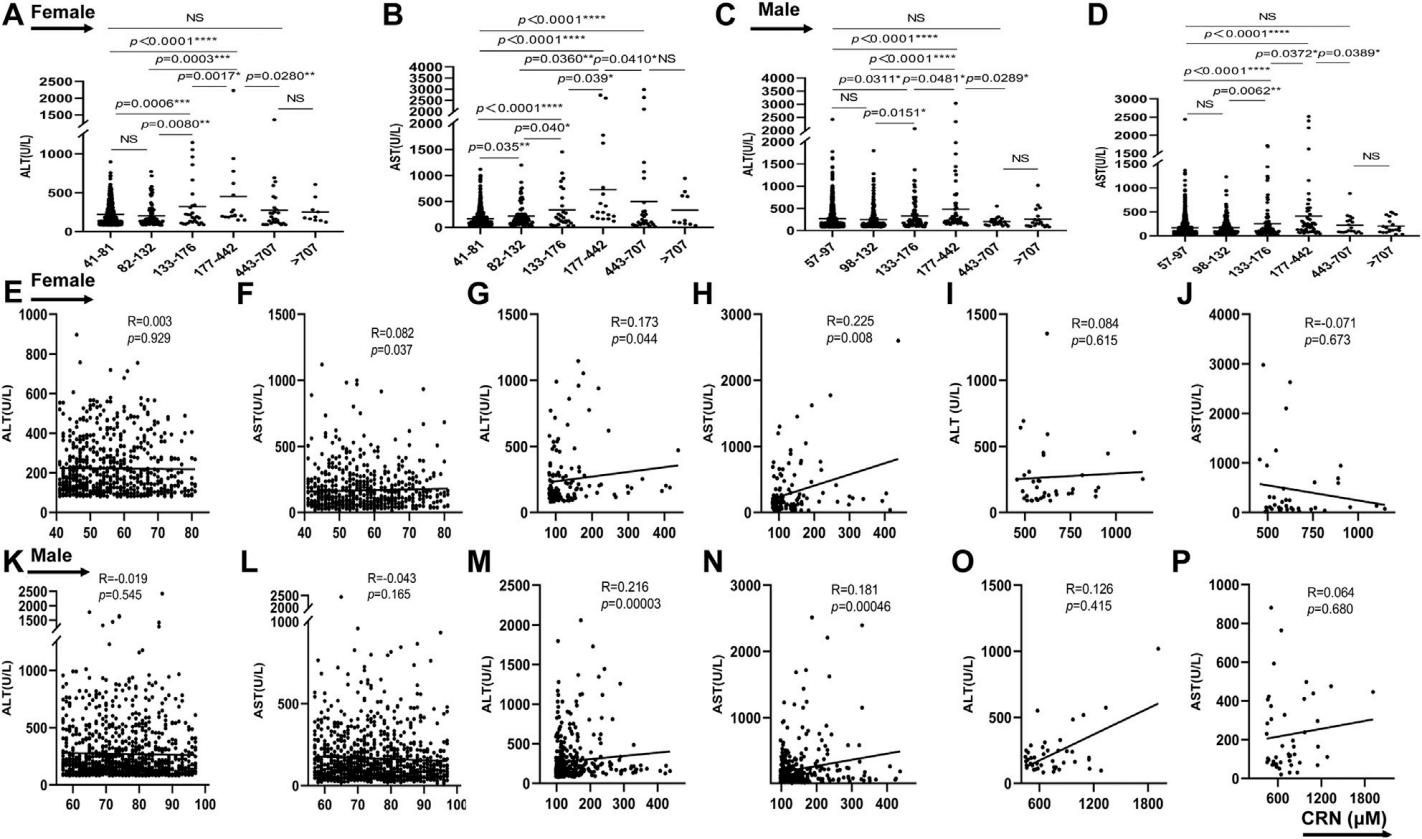
The correlation between serum creatinine concentration and ALT and AST in patients with diagnosis of drug-induced liver injury. From **(A–D)**, the serum ALT and AST levels of female and male were showed in different groups. From **(E–P)**, the relationship between ALT/AST and creatinine were analyzed in female or male patients. *p < 0.05, **p < 0.01, ***p < 0.001, ***p < 0.0001, NS, not significant.

When analysed by creatinine level, the subjects with increased creatinine in group 3 and 4 were more likely to have higher level of ALT for female and male than those with normal creatinine in group 1 (*p* = 0.0006/*p* = 0.0311 for female/male in group 1 vs. group 3; *p* < 0.0001/*p* < 0.0001 for female/male in group 1 vs. group 4). While it had no statistical difference when the creatinine is 82/98-132 and over 442 μmol/L (Figures 2A,C). As shown in Figure 2B, compared with group 1, the serum AST levels from group 2 to group 5 were increased in female (*p* = 0.035 in group1 vs. group2; *p* < 0.0001 in group1 vs. group 3, 4, and 5). Difference from female, the level of AST in male showed higher only in group 3 and group 4 than those in group 1 and showed no difference between group1 and group 2, 5, and 6 (Figure 2D). Additionally, the clinical data analysis about 590 patients with increased creatinine showed the serum ALT and AST levels were upregulated with gradual increase of creatinine from group 2 to group 4 both in female and male (*p* = 0.008/*p* = 0.04 in group 2 vs. group 3, *p* = 0.003/*p* = 0.036 in group 2 vs. group 4, *p* = 0.0017/*p* = 0.039 in group 3 vs. group 4 for female about ALT/AST; *p* = 0.0151/*p* = 0.0062 in group 2 vs. group 3, *p* < 0.0001/*p* < 0.0001 in group 2 vs. group 4, *p* = 0.0481/*p* = 0.0372 in group 3 vs. group 4 for male about ALT/AST). However, when the creatinine is over 442 μmol/L, the levels of ALT and AST were downregulated compared to group 4 and there was no statistical difference between group 5 and group 6 (*p* = 0.028/*p* = 0.041 in group 4 vs. group 5 for female about ALT and AST; *p* = 0.0289/*p* = 0.0389 in group 4 vs. group 5 for male about ALT and AST) (Figures 2A–D), which is consistent with previous report that AST and ALT values tended to be higher among patients classified as stage 3 compared with those classified as stage 4 or 5 (Sette and Lopes, 2015). As showed in Figures 2E,F further analysis in female suggested that the creatinine concentration between 41-81 μmol/L is positively correlated with AST (*p* = 0.037), but no correction with ALT. The creatinine concentration between 82–442 μmol/L in female is positively correlated with ALT and AST (*p* = 0.044 and *p* = 0.008, respectively) (Figures 2G,H). While there was no correlation between creatinine and ALT/AST when creatinine concentration is over 442 μmol/L (Figures 2I,J). Interestingly, the creatinine concentration between 57–97 μmol/L in male showed no correction with ALT/AST (Figures 2K,L). Consisting with results in female, the creatinine concentration between 98–442 μmol/L in male is also positive correlation with ALT/AST (*p* = 0.0003 and *p* = 0.00046) (Figures 2M,N). When creatinine concentration is over 442 μmol/L, it showed no correlation between creatinine and ALT/AST (Figures 2O,P). These results suggested that serum creatinine is closely related to liver function of drug-induced liver injury, but only when the creatinine concentration is between 82/98–442 μmol/L for female/male. In addition, the creatinine between 41–81 μmol/L in female was also a positive correlation with AST.

### Creatinine challenge aggravates APAP-induced liver damage in mice

**FIGURE 3:**
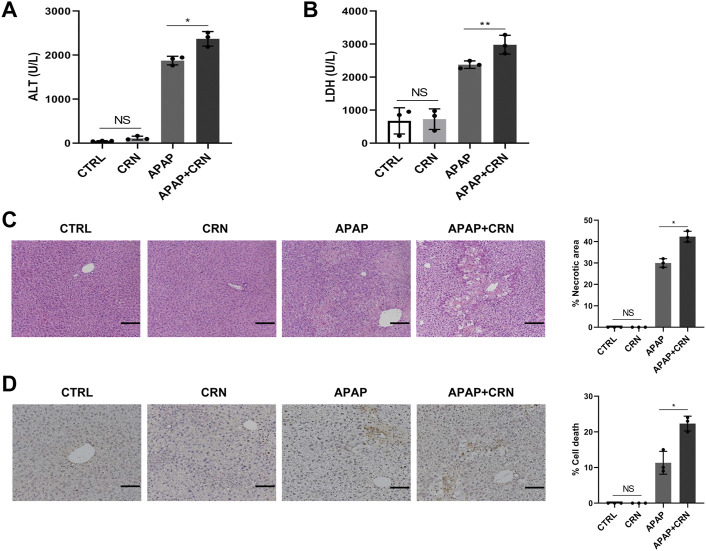
Creatinine aggravates APAP-induced liver damage in mice. Mice were pre-injected intraperitoneally with creatinine (16 mg/g) or saline for 1 week before injecting intraperitoneally with APAP (400 mg/kg) and then sacrificed after APAP-treatment for 24 h (n = 3/group). **(A,B)** The serum level of ALT and LDH were assessed. **(C)** Histological analysis of mouse livers was performed with H&E staining after APAP exposure for 24 h. **(D)** The hepatocyte death in mice liver tissues was evaluated by TUNEL staining. Scale bars = 50 μm *p < 0.05, **p < 0.01, NS, not significant. Data are representative of three independent experiments.

To further explore the impact of creatinine in APAP-induced liver damage, serum biochemistry and histopathology in mice after APAP with or without creatinine challenge were detected. The serum levels of ALT and LDH were higher in creatinine-pretreated mice than those in normal saline-administrated mice upon APAP challenge for 24 h (Figures 3A,B). Furthermore, histological analysis and the TUNEL staining together showed that liver tissues after treatment with creatinine combined with APAP exhibited severe liver damage and much more hepatocyte death than that treatment with only APAP (Figures 3C,D). Together, these data suggested that creatinine renders mice more susceptible to APAP hepatotoxicity.

### Creatinine treatment enhances the hepatic damage induced by oxidative stress in APAP-induced liver injury.

**FIGURE 4:**
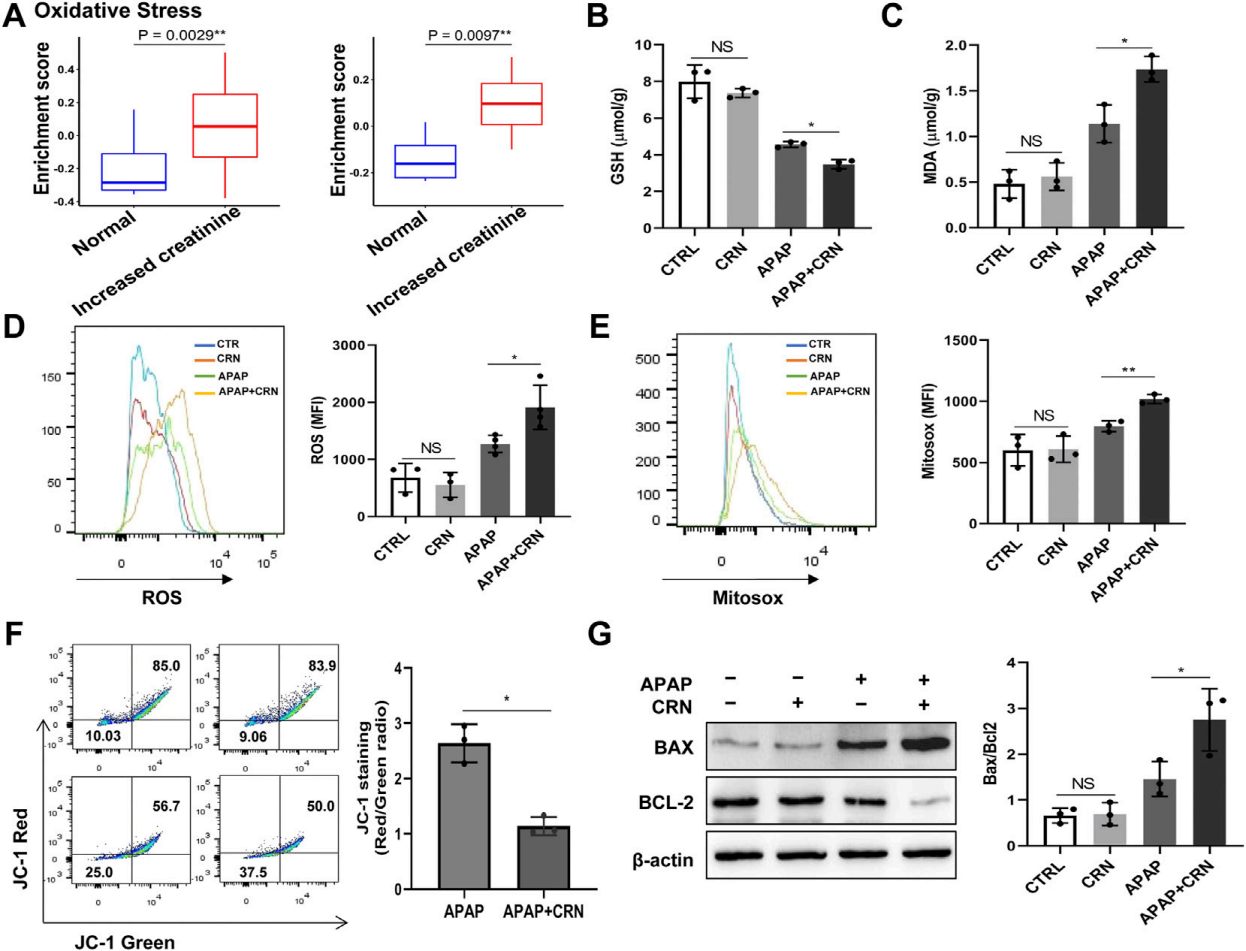
Hepatic oxidative stress level is increased after APAP exposure with creatinine treatment. **(A)** Bioinformatics analysis about oxidative stress signaling in normal creatinine (healthy people) and increased creatinine (acute kidney injury or CKD patients) from two available human dataset (left: GSE30718, right: GSE62792) was performed. After pretreatment with creatinine (16 mg/g) for 1 week, the mice were exposed to APAP (400 mg/kg) for 24 h and the GSH depletion and the MDA accumulation in liver tissue was evaluated following the instructions provided by manufacturers **(B,C)**, (*n* = 3). The generation of intracellular and mitochondria ROS were assayed after exposure to APAP for 6 h using flow cytometry **(D,E)**, (*n* = 3). **(F)** Mitochondrial membrane potential was detected by JC-1 staining after APAP challenge for 6 h (*n* = 3). **(G)** Western blotting analysis assessed the protein expression of Bax and Bcl2 in liver tissue after APAP exposure with or without creatinine pretreated for 24 h (*n* = 3). *p < 0.05, **p < 0.01, NS, not significant. Data are representative of three independent experiments.

The bioinformatics analysis about normal creatinine in healthy people and increased creatinine in acute kidney injury or CKD patients from two available human dataset (GSE30718, GSE62792) revealed that the enrichment score of oxidative stress signaling in patients with acute kidney injury or CKD were higher than that in healthy people (Figure 4A). Oxidative stress is also involved in the various toxicities associated with APAP. We focused on oxidative stress-related signaling. The GSH anti-oxidant system plays a significant role in detoxifying hepatotoxicants in APAP-induced acute liver injury. APAP challenge induced a rapid GSH depletion and excessive malondialdehyde (MDA) accumulation, thereby causing oxidative insult in the mouse liver. The data showed that creatinine challenge accelerated GSH consumption in APAP-induced liver injury (Figure 4B). Besides, the hepatic level of MDA was significantly higher in creatinine-pretreated mice than that in control mice after APAP administration (Figure 4C). Meanwhile, the intracellular and mitochondrial ROS levels in liver cells were measured by flow cytometry, respectively (Figures 4D,E). Moreover, primary mouse hepatocytes were isolated and a decreased JC-1 red/green fluorescence ratio in hepatocytes from mice pretreated with creatinine than those from mice treated with APAP was measured (Figure 4F). The expression of Bax and Bcl2 is considered a critical role in underlying APAP-induced hepatotoxicity. Herein, enhanced Bax level and downregulated Bcl2 expression were observed in liver tissue from mice treated with APAP combined with creatinine compared with mice treated with APAP alone (Figure 4G). Collectively, these results suggested that creatinine treatment enhances the hepatic damage in APAP-induced liver injury by promoting oxidative stress responses.

### ROS inhibition can rescue aggravated liver damage caused by creatinine after APAP exposure

**FIGURE 5:**
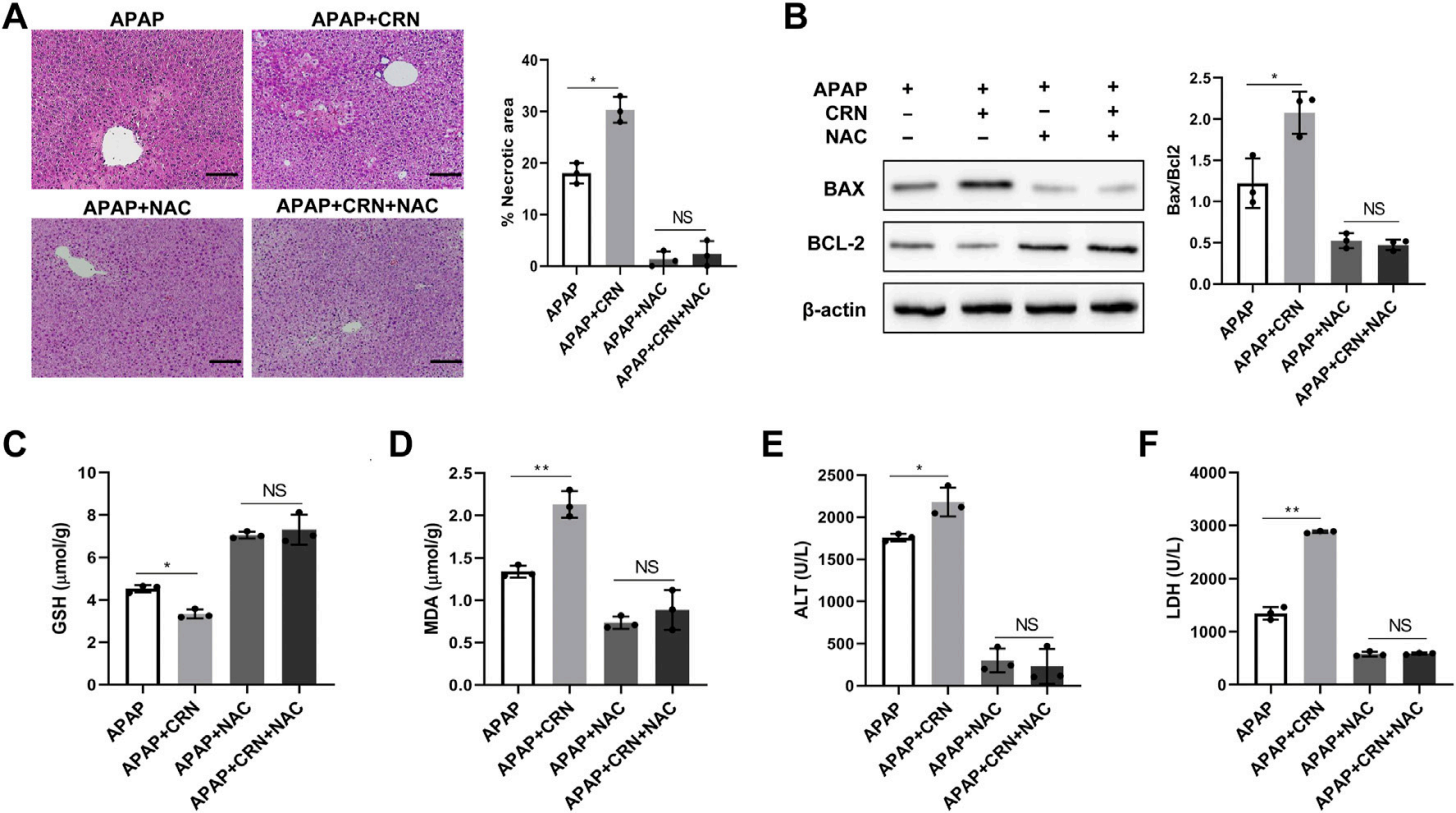
ROS inhibition rescues aggravated APAP-induced liver injury by creatinine.After 1 week pretreated with creatinine, mice were treated with 150 mg/kg of NAC 1 h before APAP challenge for 24 h (*n* = 3/group). **(A)** Histological analysis was exploited to assess the severity of liver injury. **(B)** The expression of Bax and Bcl2 in liver tissue were evaluated by Western Blotting. **(C–F)** The biochemical indicators including GSH and MDA in liver tissue and ALT and LDH in serum were determined after inhibition ROS. Scale bars = 50 µm *p < 0.05, **p < 0.01, NS, not significant. Data are representative of three independent experiments.

The above results revealed creatinine enhances the hepatic damage through ROS accumulation in APAP-induced liver injury. Therefore, a well-known anti-oxidant, NAC was used to block the ROS and evaluate the pathophysiological role of ROS in creatinine regulating APAP-related liver injury. Notably, the severity of liver damage was reduced and comparable between APAP-injected mice treated with or without creatinine by pretreating with NAC (Figure 5A). Additionally, the hepatic levels of Bax and Bcl2 were similar between APAP-treated mice with or without creatinine administration after NAC treatment (Figure 5B). Also, hepatic levels of GSH and MDA were comparable between APAP-injected mice in the presence or absence of creatinine mice when ROS was inhibited (Figures 5C,D). The biochemical analysis determined that the sera from creatinine-pretreated mice and control mice in response to APAP showed no significant difference in the levels of ALT activity and LDH activity when ROS was blocked (Figures 5E,F). Collectively, the results confirmed that creatinine aggravates APAP-related liver injury by oxidative stress.

### Creatinine treatment exacerbates APAP-induced liver injury through activation of the ROS/JNK signaling pathway

**FIGURE 6:**
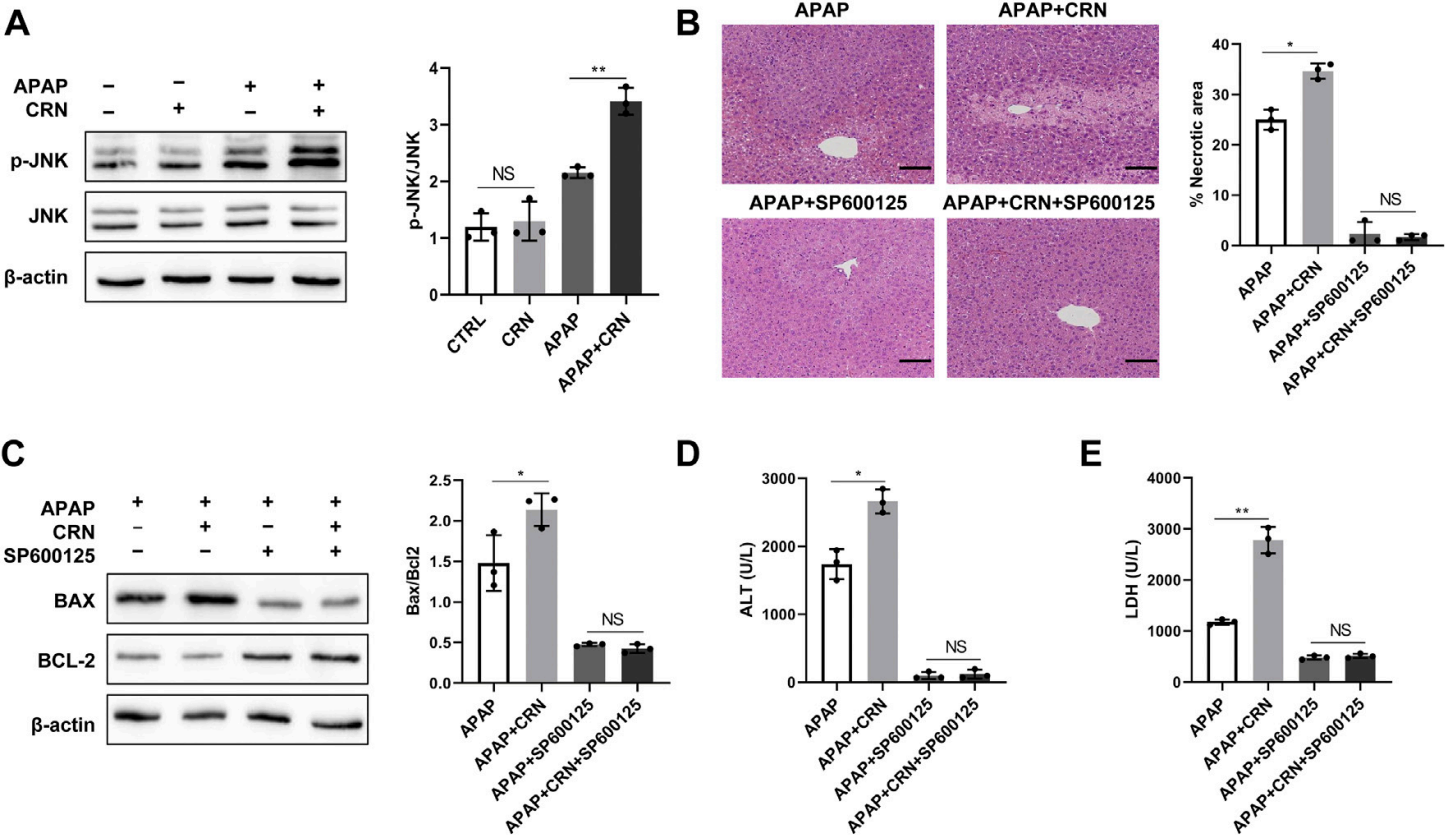
Creatinine exacerbates APAP-induced liver injury through activation of JNK. **(A)**Mice were pre-injected intraperitoneally with creatinine for 1 week and then exposure to APAP (400 mg/kg) for 24 h (*n* = 3/group). Western blotting analysis was used to detect the JNK phosphorylation. From figure **(B–E)**, mice were injected intraperitoneally with the JNK inhibitor SP600125 1 h prior the APAP injection. **(B)** H&E staining was performed to analyse the mouse liver injury after blocking JNK. **(C)**The protein level of Bax and Bcl2 were determined using western blotting. **(D,E)** The ALT and LDH activities in serum were measured. Scale bars = 50 µm *p < 0.05, **p < 0.01, NS, not significant. Data are representative of three independent experiments.

ROS generation in APAP-induced liver injury can activate JNK, thus initials hepatocyte cell death. The role of the JNK pathway in creatinine-mediated exacerbation of APAP-induced liver injury was investigated. The results showed that APAP induced a strongly higher level of JNK phosphorylation in livers from creatinine pre-treated mice than those from control mice (Figure 6A). Moreover, the specific JNK inhibitors, SP600125, could rescue mice from APAP-induced liver injury and eliminate the differences between APAP plus creatinine-treated mice and only APAP-treated mice (Figure 6B). The hepatic levels of Bax and Bcl2 were similar between APAP-treated mice with or without creatinine administration after JNK inhibition (Figure 6C). Biochemical analyses showed that the sera from creatinine-pretreated mice and control mice in response to APAP showed no significant difference in the activities of ALT and LDH when JNK activation was blocked (Figures 6D,E). Together, these data suggested that creatinine accelerates the development of APAP-induced liver injury by inducing JNK activation.

### Creatinine increases APAP-induced cell death in human HepaRG cells

**FIGURE 7:**
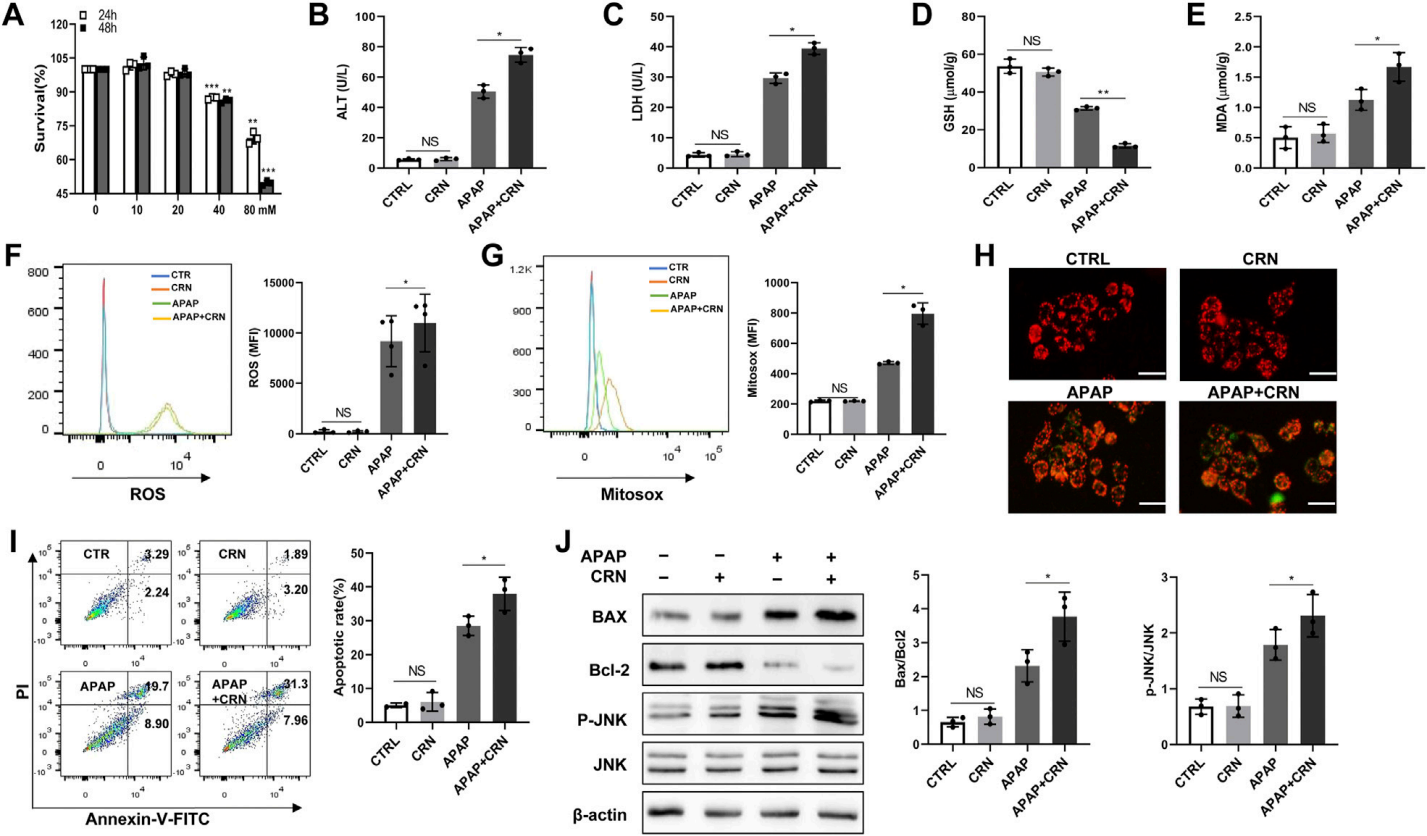
Creatinine enhances APAP-induced cell death in human HepaRG cells by ROS/JNK signaling pathway. **(A)** The HepaRG cells were stimulated with different concentration of creatinine for 24 or 48 h to detect the drug toxicity using CCK8. From **(B–J)**, the cells were administrated with 20 mM APAP accompanying with or without 20 mM creatinine for 24 h **(B,C)** The ALT and LDH release in HepaRG cells were determined. **(D,E)** The intracellular level of GSH and MDA were assayed. **(F,G)** The ROS generation inside the cell and mitochondria were analyzed by flow cytometry labeling with fluorescent probe DCFH-DA and Mitosox following exposure to APAP for 6 h. **(H)** Mitochondrial membrane potential was detected by JC-1 staining after APAP challenge for 6 h using immunofluorescence. **(I)**The cell death was determined by flow cytometry at 6 h post- APAP administration. **(J)**Western blotting was used to analyze the phosphorylation of JNK, Bcl2 and Bax after APAP challenge for 24 h *p < 0.05, **p < 0.01, NS, not significant. Data are representative of three independent experiments.

So far, all the *in vivo* studies demonstrated that creatinine could aggravate APAP-induced liver damage through activating ROS/JNK signaling pathway. Human HepaRG cells were stimulated with APAP in the presence or absence of creatinine to evaluate the effect of creatinine in APAP-stimulated HeapRG cells. Here, different concentrations of creatinine (0, 10, 20, 40, and 80 mM) was used to assess cytotoxicity in HepaRG by CCK8 assay (Figure 7A). Then 20 mM creatinine concentration, which showed no detrimental cytotoxicity in the HepaRG and reported to inhibit the replication of *S. aureus* (Smithee et al., 2014), was used to further substantiate the effect of creatinine on APAP-induced liver damage. Compared with the cells treated with APAP alone, the HepaRG treated with APAP plus creatinine produced increased levels of ALT and LDH (Figures 7B,C). Besides, decreased GSH level while elevated MDA production was found in cells treated with APAP combined with creatinine compared to the cells treated with APAP only (Figures 7D,E). Moreover, the intracellular and mitochondrial ROS levels were increased in the creatinine plus APAP treated cells than that in APAP-treated cells (Figures 7F,G). Then the downregulated membrane potential was observed in HepaRG cells challenged with APAP plus creatinine compared to the cells treated with APAP alone, as determined by JC-1 dye staining (Figure 7H). Notably, more apoptotic cells were exhibited in HepaRG cells treated with creatinine plus APAP than those treated with APAP only (Figure 7I). Additionally, increased Bax and reduced Bcl2 were found in HepaRG cells treated with APAP combined with creatinine (Figure 7J). APAP-stimulated HepaRG cells exhibited much more phosphorylation of JNK in the presence of creatinine (Figure 7J). Taken together, the data indicated that creatinine can potentiate APAP-induced hepatocyte death through ROS/JNK signaling pathway.

## Discussion

The serum creatinine is widely interpreted as a measure of the GFR and a novel biomarker for CKD (Perrone et al., 1992; Cánovas et al., 2019). APAP is the most available analgesic and antipyretic medication, which is also as a clinical pharmacology considerations for pain management in patients with kidney diseases (Koncicki et al., 2017). However, APAP overdose is the most frequent cause of acute liver failure in the United States and many other countries (Lee, 2013). The role of creatinine in APAP-induced liver injury should be clarified to guide APAP usage in renal patients. In the present study, it suggested that serum creatinine levels were correlated with the levels of ALT and AST in drug-induced liver injury. Creatinine aggravated APAP hepatotoxicity, as evidenced by severe hepatocyte death as well as elevated serum LDH and ALT levels in mice after intraperitoneal injection with creatinine and APAP. The mechanistic studies indicated that creatinine treatment promoted ROS production and JNK activation, thereby promoting the APAP-induced liver damage. It may provide new insight into the usage of APAP in patients with kidney problems.

Current evidence showed that creatinine could be a critical index of model for end-stage liver disease (MELD) and hepatorenal syndrome (HRS) to assess liver disease severity and mortality risk (Kamath et al., 2001; Belcher et al., 2015). A cross-sectional analysis also revealed that NAFLD subjects have higher serum creatinine than those without NAFLD (Niu et al., 2021). Elevated serum uric acid-to-creatinine ratio (SUA/Cr) may be a reliable marker for predicting NAFLD (Seo and Han, 2021). However, low serum concentrations of creatinine were observed in severe hepatic failure patients like fulminant hepatitis (Takabatake et al., 1988). Some reports showed that the creatinine-lactate score could predict the mortality of acute liver failure in patients listed for liver transplantation (Figueira et al., 2021). While the role of creatinine in drug-induced liver injury is unclear. The clinical data on drug-induced liver injury in present study demonstrated that patients with elevated creatinine, especially those with creatinine between 133–442 μmol/L, were more likely to have higher level of ALT and AST than subjects with normal creatinine. Creatinine ranging from 82/98 μmol/L to 442 μmol/L was positively correlated with ALT and AST. However, when the creatinine is over 442 μmol/L, the level of ALT and AST tended to decrease and it showed no correlation between creatinine and ALT/AST.

APAP toxicity comprises multi-stages and multi-signaling pathways, including APAP metabolism, oxidative stress, endoplasmic reticulum (ER) stress, autophagy, sterile inflammation, microcirculatory dysfunction, and compensatory liver regeneration. Many studies indicated that oxidative stress is involved in the various toxicities associated with APAP. Mitochondrial oxidative stress is the predominant cellular event in APAP-induced liver injury (Yan et al., 2018). Overdose of APAP was metabolized into NAPQI, which can deplete GSH and subsequently increase H_2_O_2_ release from mitochondria to the cytoplasm. The GSH is an important anti-oxidant, whereas MDA indirectly reflects the severity of damage induced by free radicals and is an important biomarker of oxidative damage (Halder et al., 2018). The GSH consumption and MDA accumulation are exhibited in oxidative stress-related diseases, including APAP-induced liver injury (Wu et al., 2019). Indeed, the mechanism underlying APAP-induced hepatotoxicity in humans and mice involves intracellular and mitochondrial damage and nuclear DNA fragmentation. Importantly, NAC, a known scavenger of reactive oxygen species, can really rescue the liver injury induced by APAP (Adil et al., 2018). Consistent with these studies, the results in this study showed that creatinine accelerates GSH depletion and MDA accumulation, accompanied by ROS generation. Most strikingly, blocking the ROS using NAC can ameliorate the aggravated liver injury induced by APAP plus creatinine. The mechanistic study showed that ROS/JNK signaling pathway is responsible for the creatinine-mediated exacerbation of APAP-induced liver damage. It revealed that creatinine is pivotal for oxidative stress-induced hepatocyte death during APAP-induced liver injury.

ROS generation resulting from GSH depletion in response to APAP can activate JNK signaling pathway (Win et al., 2016). Sustained activation of JNK plays an essential role in APAP-induced liver damage. It induces mitochondrial permeability transition, inhibits mitochondria bioenergetics, amplifies mitochondrial ROS, and consequently leads to the death of hepatocytes (Hanawa et al., 2008). Permeabilization of the mitochondrial outer membrane is a crucial step in the intrinsic apoptosis pathway. This process can be regulated by Bcl-2 family proteins, which can be either pro-or anti-apoptotic proteins (Hengartner, 2000; Oz and Chen, 2008; Gillies and Kuwana, 2014). Previous studies suggested that activation of JNK can cause Bax activation and translocation to mitochondria and then trigger the opening of the mitochondrial permeability transition and then release of mitochondrial intermembrane proteins, like apoptosis-inducing factor, which leads to DNA fragmentation and necrosis (Gunawan et al., 2006; Kim et al., 2006; Wang et al., 2018). In present study, the results showed that creatinine significantly enhanced the APAP-induced phosphorylation of JNK. Interestingly, SP600125, a classical inhibitor of JNK (Latchoumycandane et al., 2007), abrogated the increased Bax expression and reduced bcl2 expression in hepatocytes induced by creatinine plus APAP stimulation. All of these data indicated that creatinine may play its destructive factor in APAP-induced acute liver injury through activation of JNK signaling pathway.

Apoptosis is identified as a dominant mode of cell death in liver diseases, like alcoholic/non-alcoholic liver disease, viral hepatitis, and hepatocellular carcinoma (Guicciardi and Gores, 2005). Whether APAP-induced hepatocyte death is apoptosis-dependent is controversial. Some researchers regarded although APAP-induced hepatic cells death shares some features of apoptosis, apoptotic cell death was ruled out for the absence of caspase activation and lack of protection by caspase inhibitors and missing morphological characteristics of apoptotic cells (Jaeschke and Ramachandran, 2020). As showed in apoptosis ratio detected by flow cyctometer in HepaRG cells, APAP plus creatinine treatment induced a more significant increase in late apoptotic cells than early apoptotic cells compared with only APAP exposure. The changes of Bax and Bcl2 were also observed in responses to APAP challenge in the presence or absence of creatinine. These results indicated creatinine modulates the cellular apoptosis during APAP induced oxidative stress mediated hepatocyte toxicity.

Notably, there are several limitations in this study. First, the clinical data belongs to in-hospital desensitization text data. The potential variates including detailed treatment process and different hepatotoxic drugs usage, etc. cannot be controlled. Second, we here pay more attention to the relation between creatinine and ALT/AST, more liver function indicators such as albumin, alkaline phosphatase, etc. can be studied in the future. Finally, the number of subjects with increased creatinine is limited, which may result in a weak correlation between creatinine and ALT and AST. Therefore, prospective studies are also needed to determine whether creatinine contributes to the development of APAP-induced liver injury.

In conclusion, the study demonstrated that creatinine is closely related to ALT and AST in drug-induced liver injury. Creatinine aggravates APAP-induced liver injury through increasing ROS generation, resulting in enhanced JNK activation and thus leading to the exacerbated liver injury. The study uncovered the function of creatinine in APAP hepatotoxicity and implicated the importance of toxicity management of acetaminophen in patients with kidney diseases.

## Data Availability

The datasets presented in this study can be found in online repositories. The names of the repository/repositories and accession number(s) can be found in the article/Supplementary Material.

## Abbreviations

AST, Aspartate aminotransferase; ALT, Alanine aminotransferase; TBIL, Total Bilirubin; ALB, Albumin; ALP, Alkaline phospatase.
