# Supplementary figures and images for: Creatinine accelerates APAP-induced liver damage by increasing oxidative stress through ROS/JNK signaling pathway
Source: Front Pharmacol. 2022 Aug 24;13:959497. doi: 10.3389/fphar.2022.959497 (PMC9449354; doi:10.3389/fphar.2022.959497)

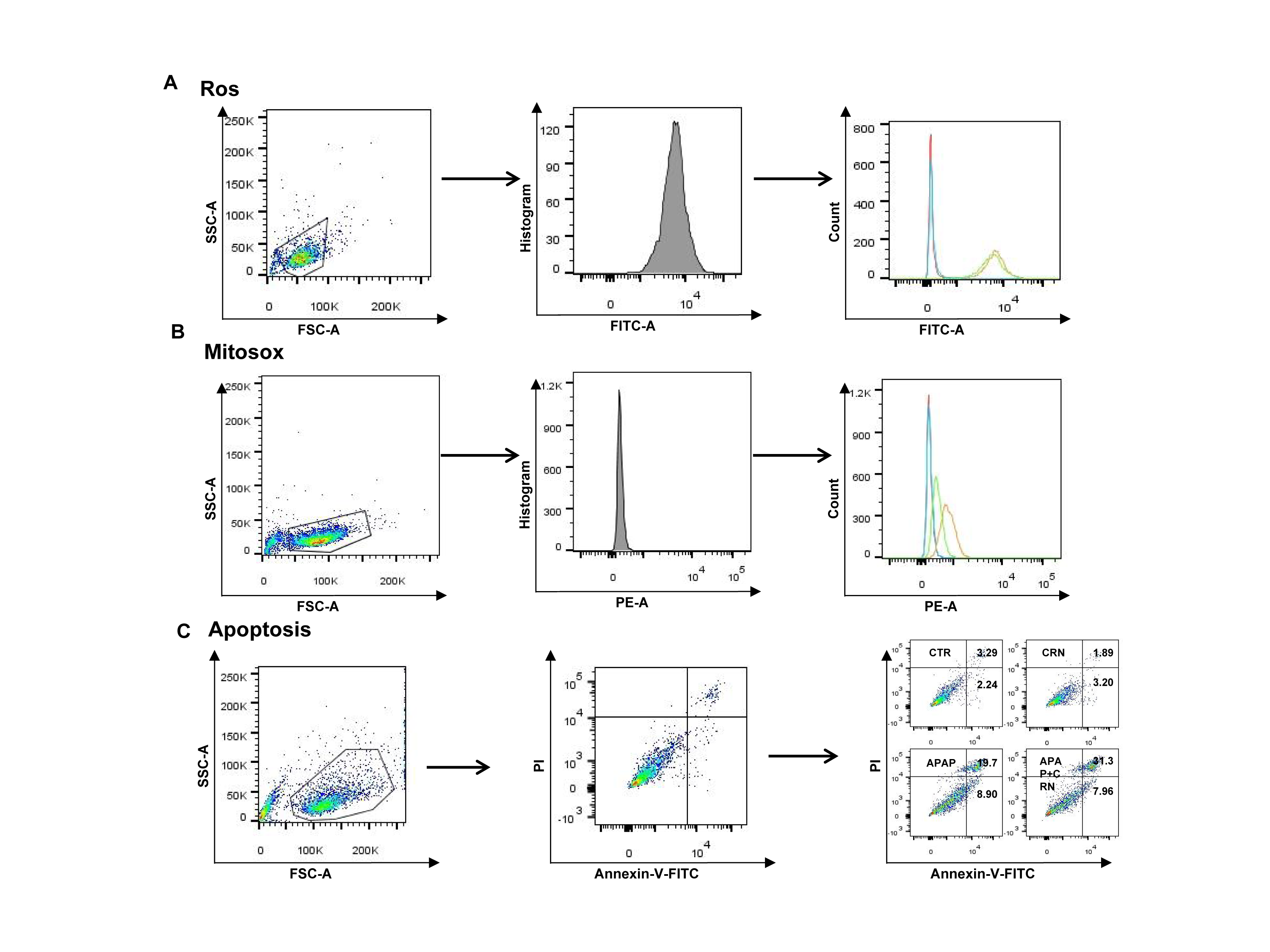

Supplement: Supplementary file 2 [file Image6.TIF]

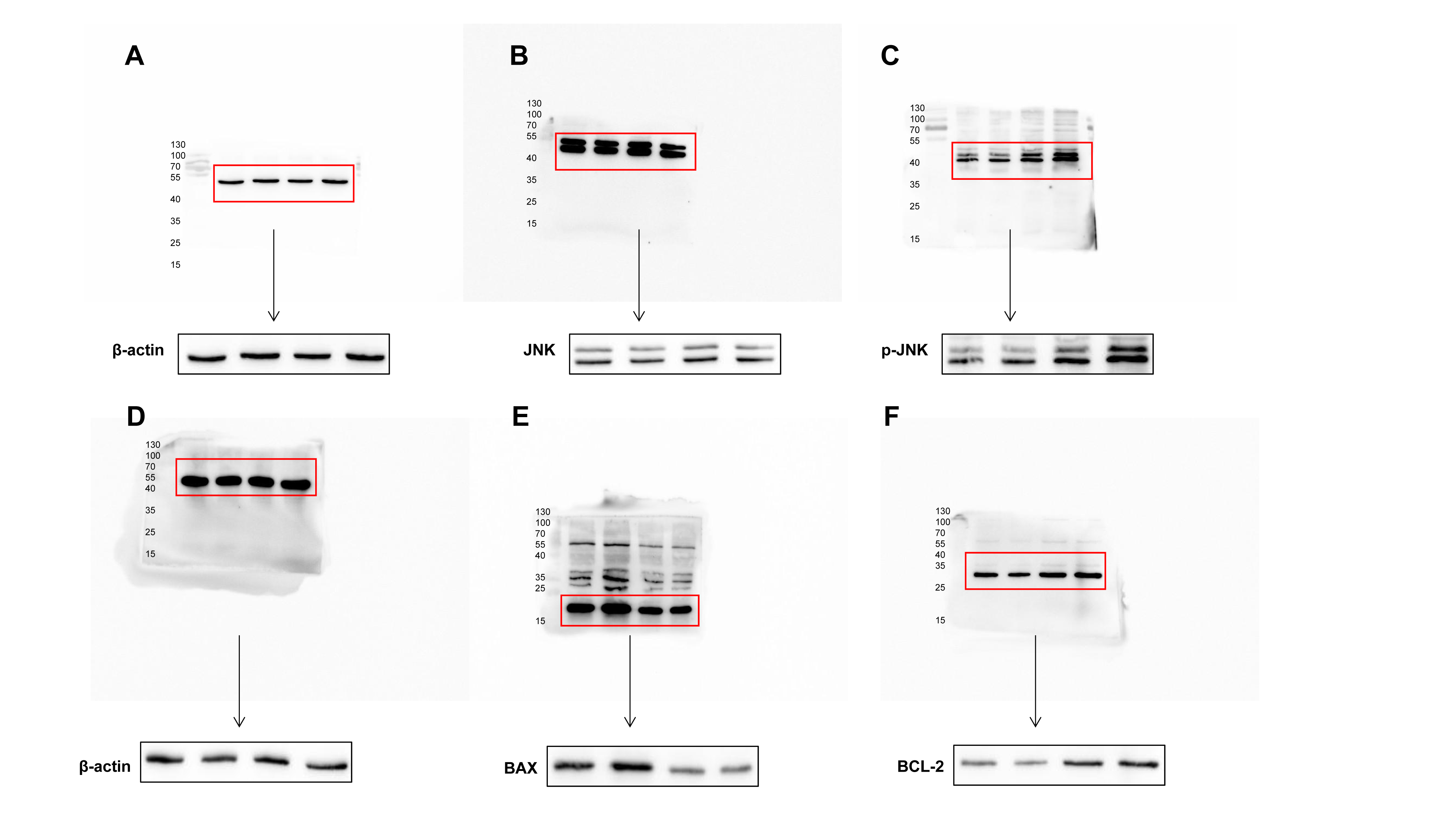

Supplement: Supplementary file 5 [file Image3.TIF]

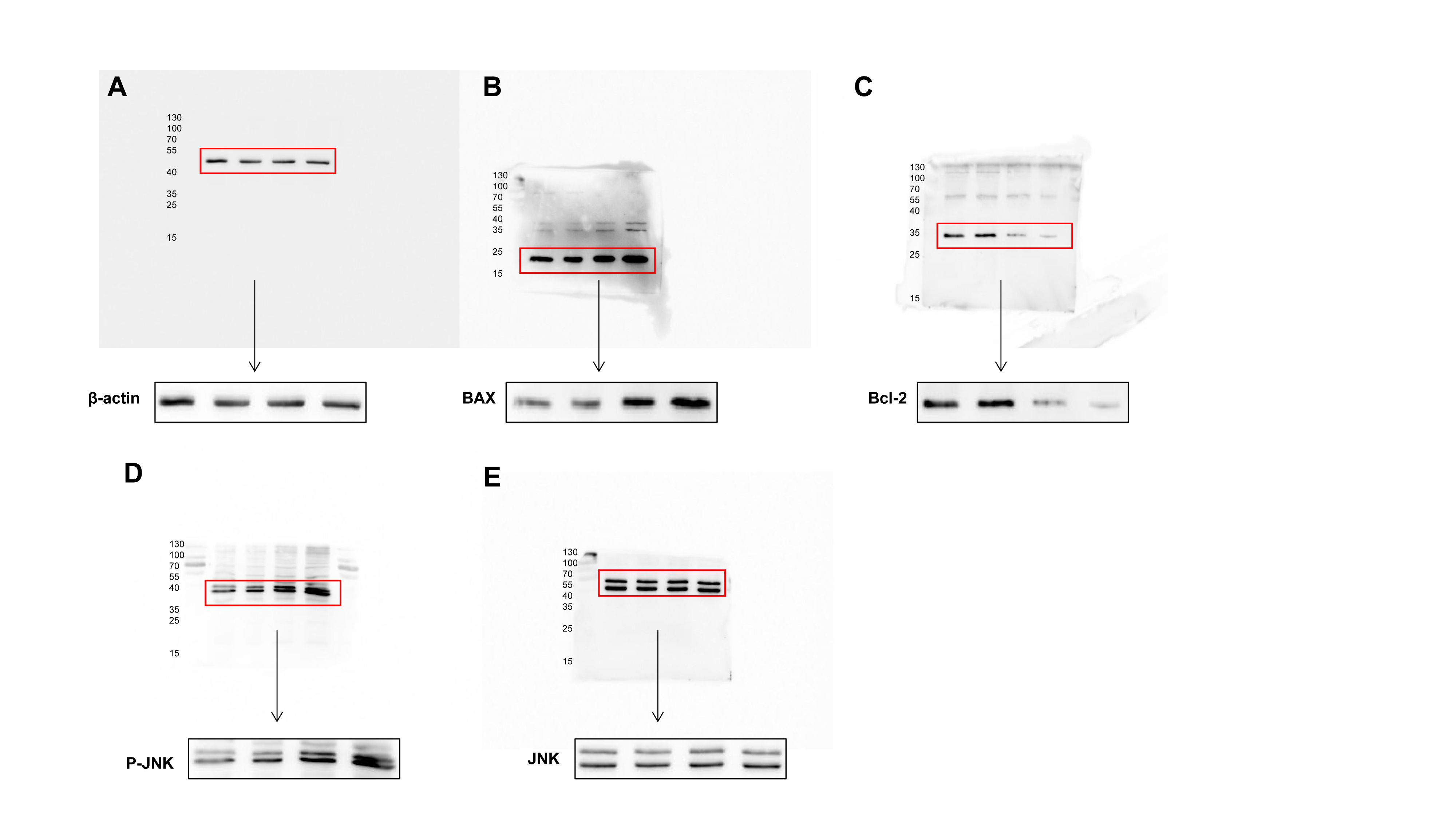

Supplement: Supplementary file 6 [file Image4.TIF]

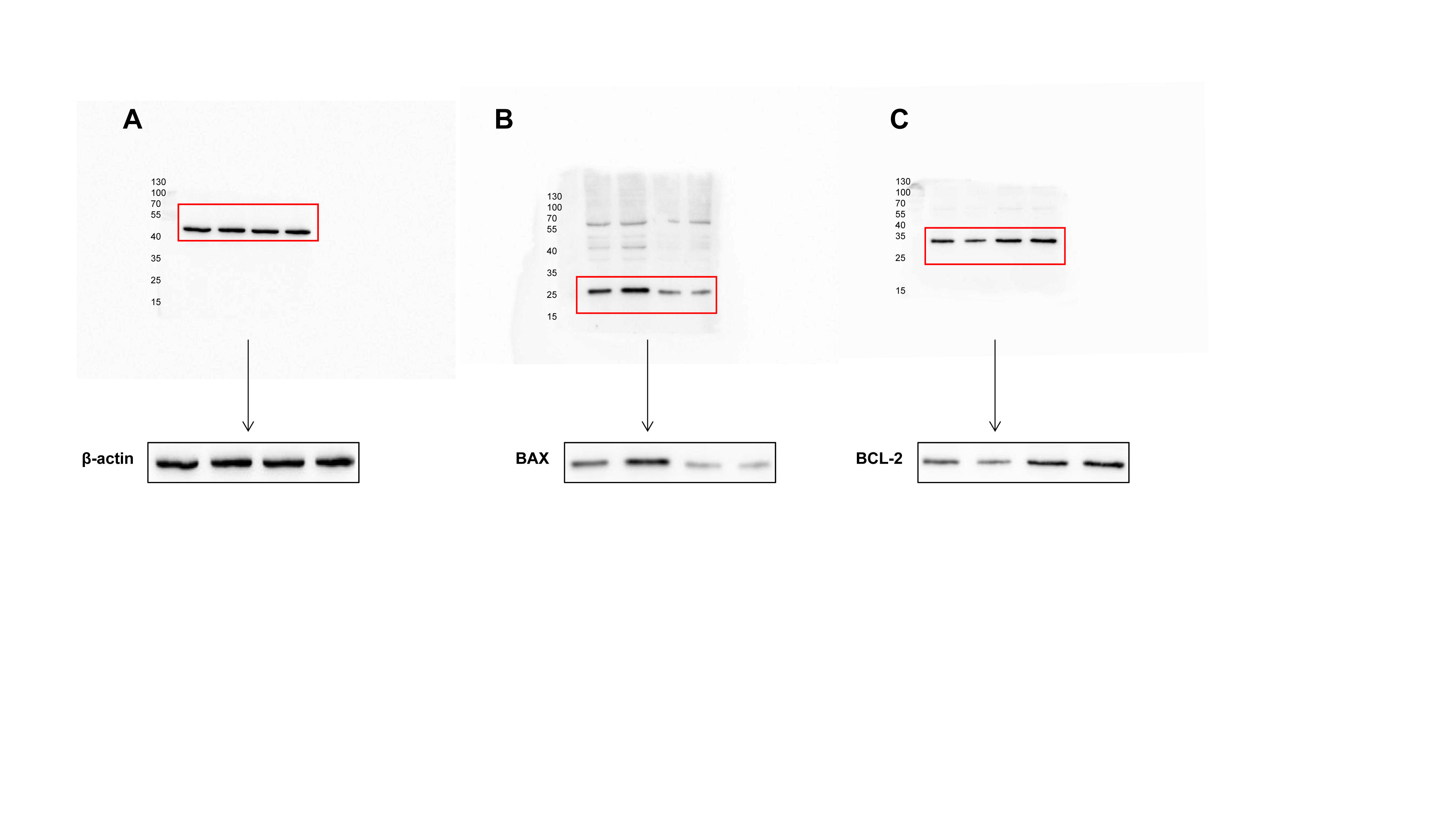

Supplement: Supplementary file 8 [file Image2.TIF]

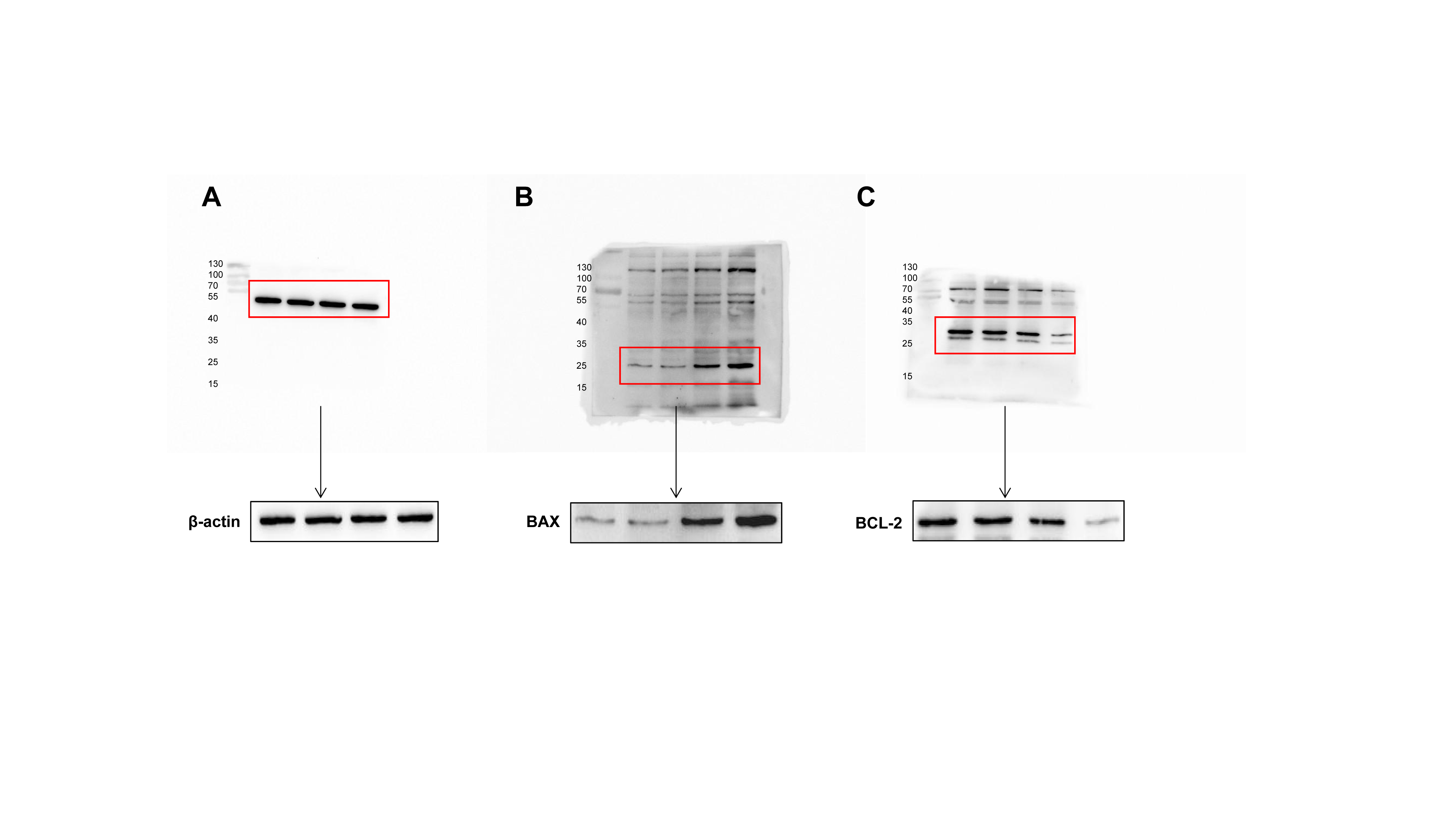

Supplement: Supplementary file 9 [file Image1.TIF]

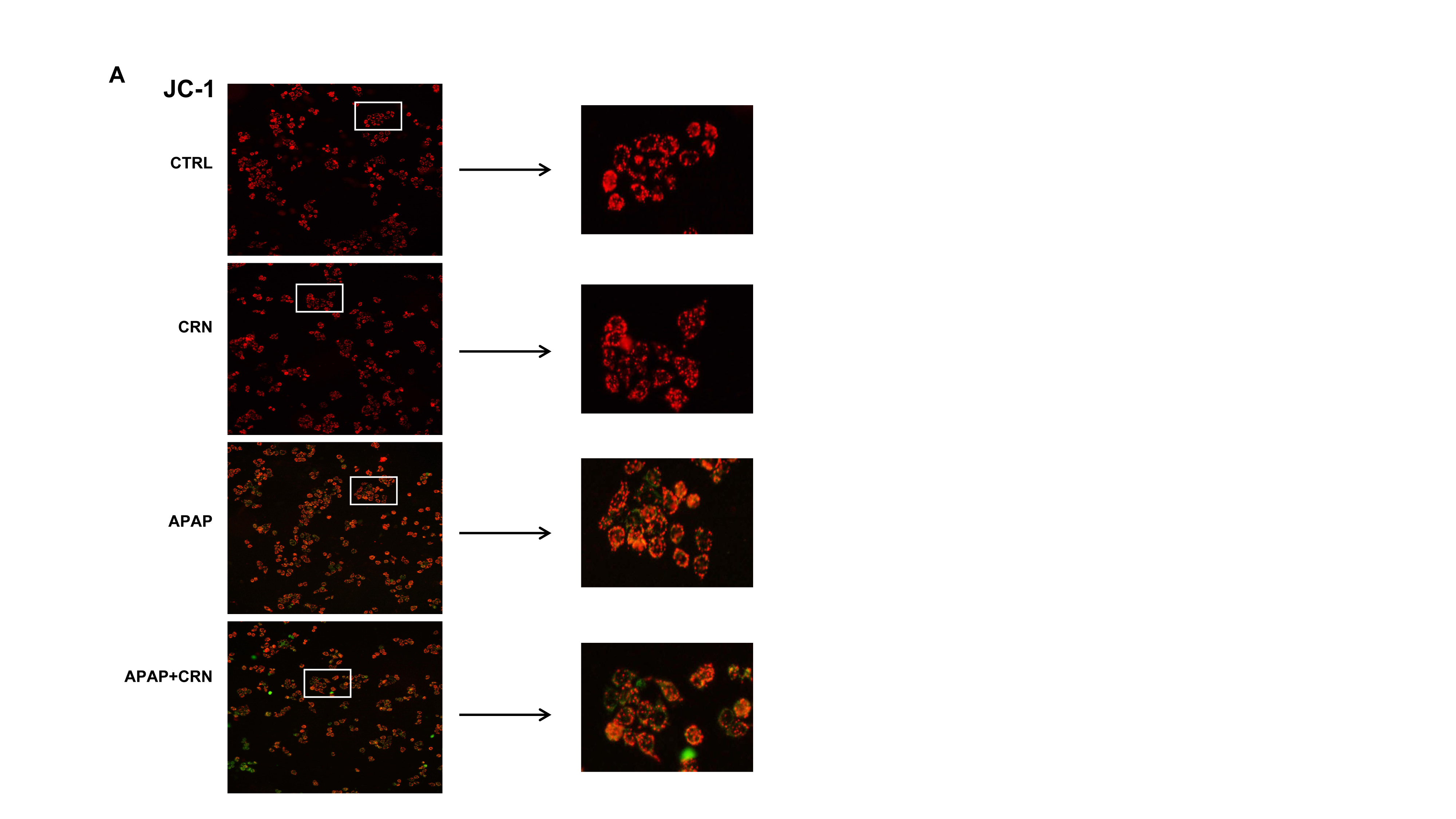

Supplement: Supplementary file 10 [file Image7.TIF]

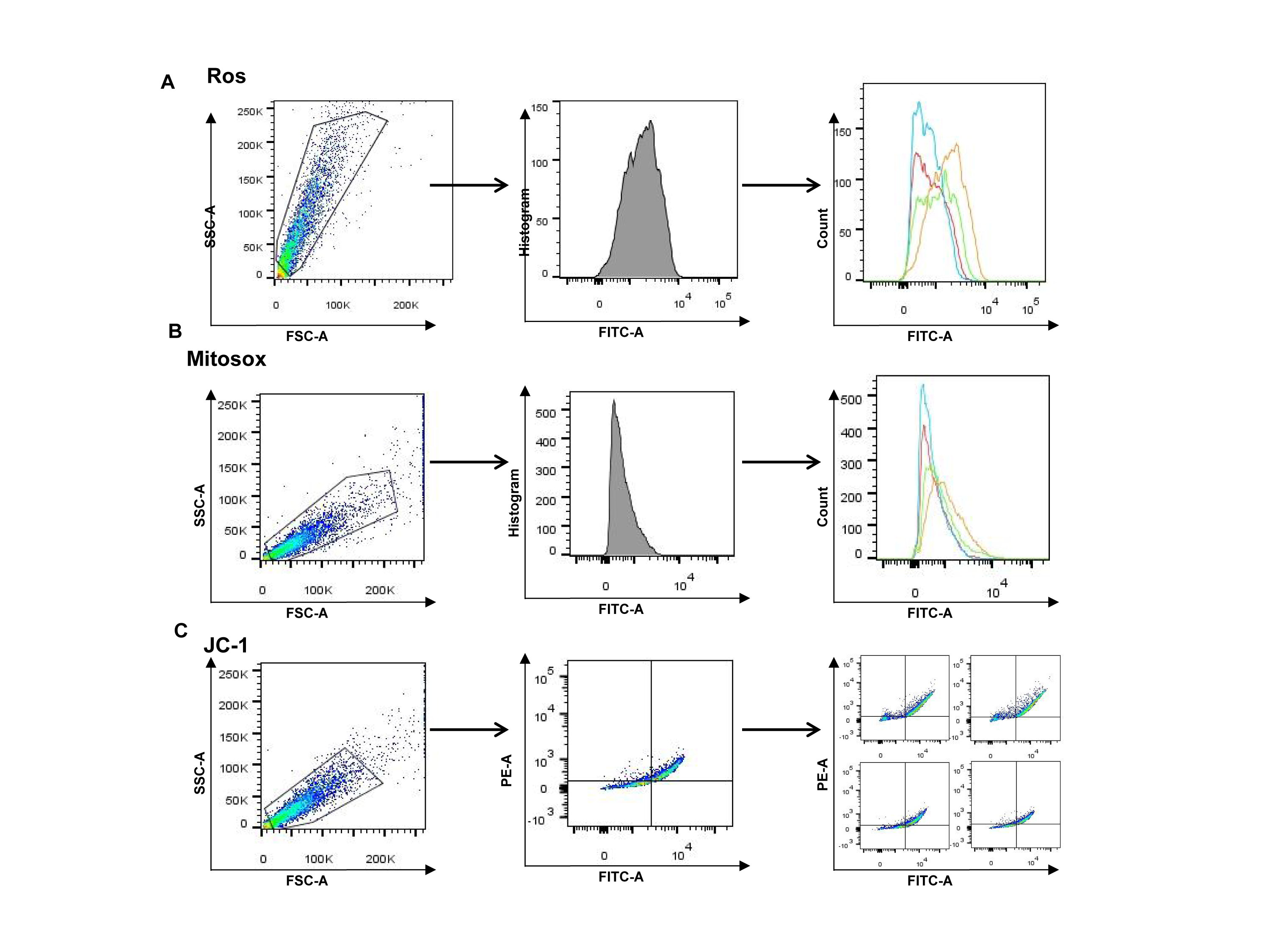

Supplement: Supplementary file 11 [file Image5.TIF]
