# Supplementary material for: Creatinine accelerates APAP-induced liver damage by increasing oxidative stress through ROS/JNK signaling pathway
Source: Front Pharmacol. 2022 Aug 24;13:959497. doi: 10.3389/fphar.2022.959497 (PMC9449354; doi:10.3389/fphar.2022.959497)
Supplement: Supplementary file 3 [file Table1.docx]

**Table 1.**

**Main baseline clinical data**

| Variables | Female | Male |
| --- | --- | --- |
| Age (years) | 40.96±16.38 | 41.76±16.54 |
| gender(%) | 36%(831/2282) | 64%(1451/2282) |
| Creatinine (μmol/L) | 96.10±133.41 | 113.90±132.99 |
| AST (U/L) | 200.63±264.22 | 182.17±225.95 |
| ALT (U/L) | 228.15±160.05 | 272.77±253.29 |
| TBIL(μmol/L) | 80.92 ±125.49 | 101.02±159.33 |
| ALB (g/L) | 34.34±5.99 | 34.72±6.40 |
| ALP (mmol/L) | 185.22±186.41 | 163.67±107.63 |

Abbreviations：AST Aspartate aminotransferase， ALT Alanine aminotransferase，TBIL Total Bilirubin，ALB Albumin, ALP Alkaline phospatase
