# Supplementary material for: Creatinine accelerates APAP-induced liver damage by increasing oxidative stress through ROS/JNK signaling pathway
Source: Front Pharmacol. 2022 Aug 24;13:959497. doi: 10.3389/fphar.2022.959497 (PMC9449354; doi:10.3389/fphar.2022.959497)
Supplement: Supplementary file 4 [file Presentation1.PPTX]

## Slide 1
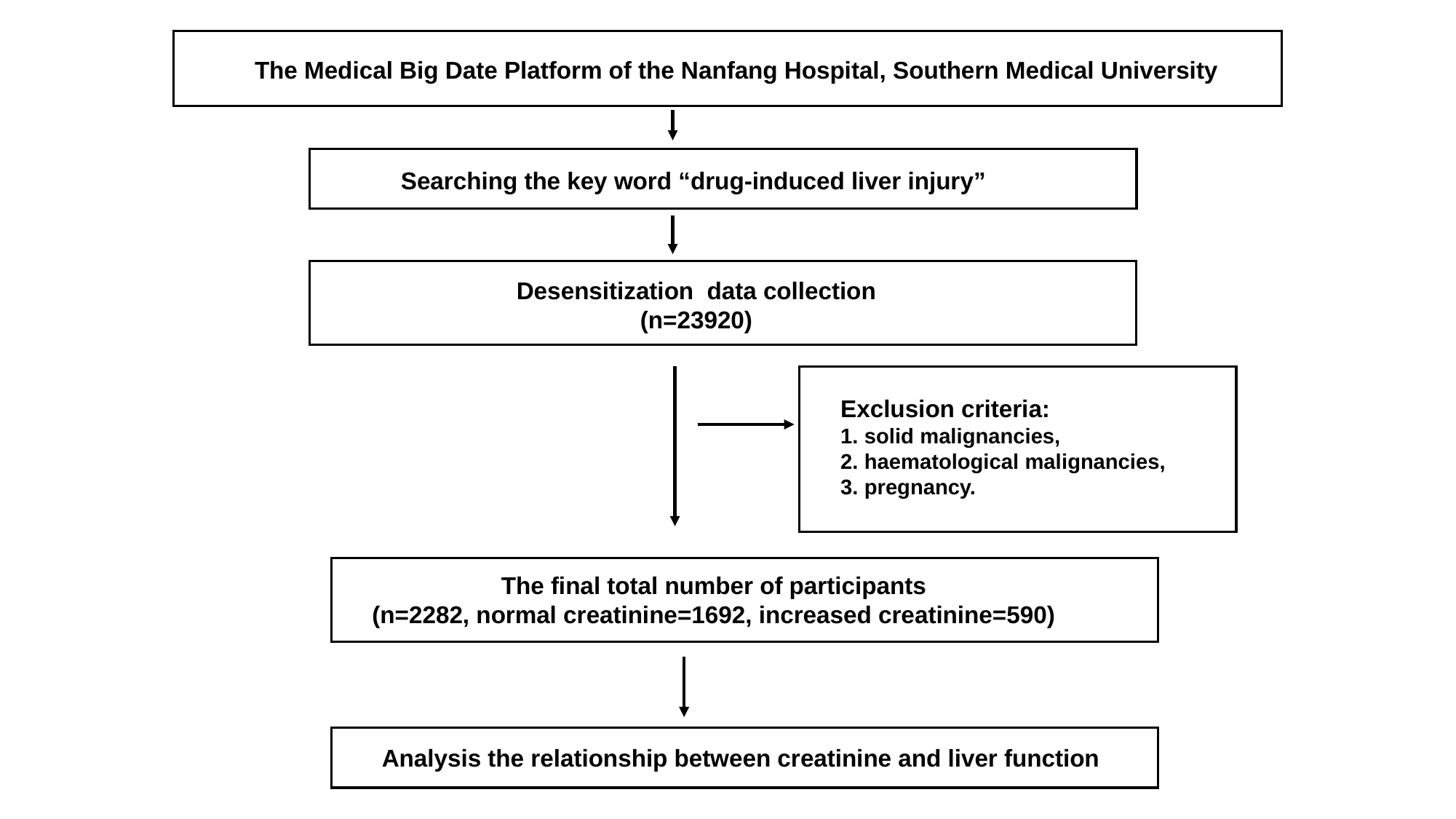

The Medical Big Date Platform of the Nanfang Hospital, Southern Medical University
Searching the key word “drug-induced liver injury”
Desensitization data collection
(n=23920)
Exclusion criteria:
1. solid malignancies,
2. haematological malignancies,
3. pregnancy.
The final total number of participants
(n=2282, normal creatinine=1692, increased creatinine=590)
Analysis the relationship between creatinine and liver function

## Slide 2
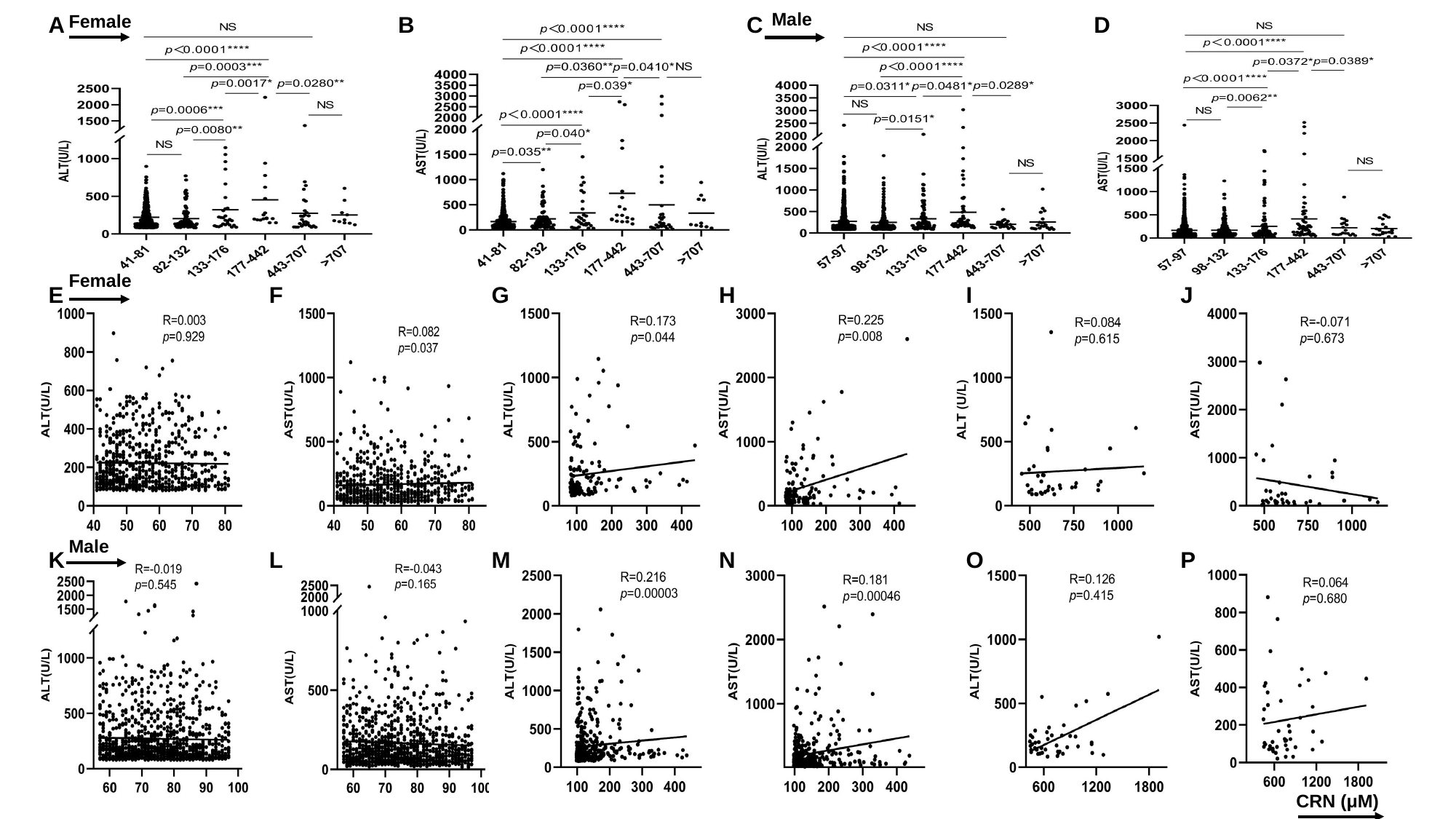

Male
A
Female
B
C
D
Female
E
F
G
H
I
J
Male
K
L
M
N
O
P
CRN (μM)

## Slide 3
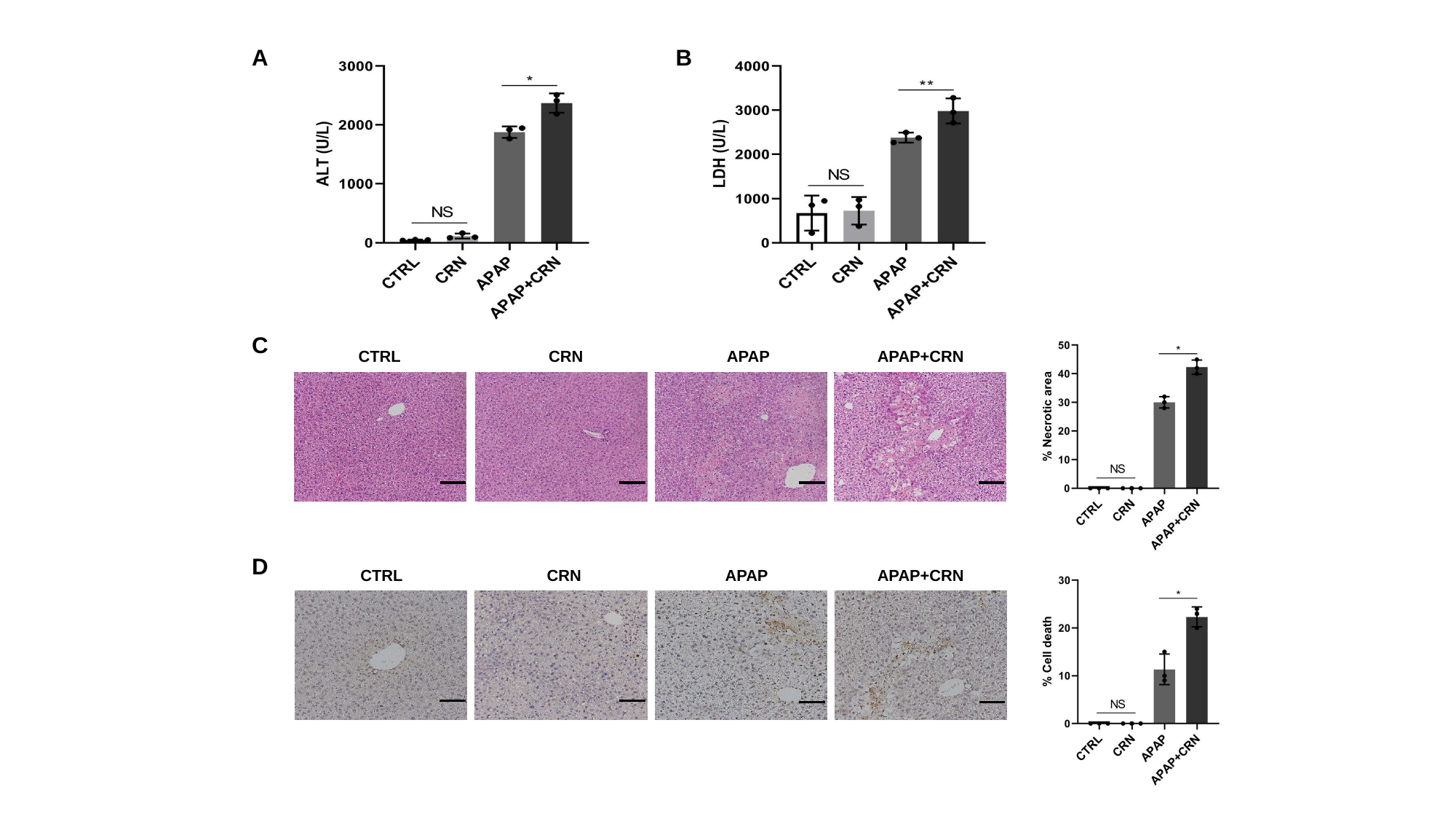

A
B
C
CTRL
CRN
APAP
APAP+CRN
D
CTRL
CRN
APAP
APAP+CRN

## Slide 4
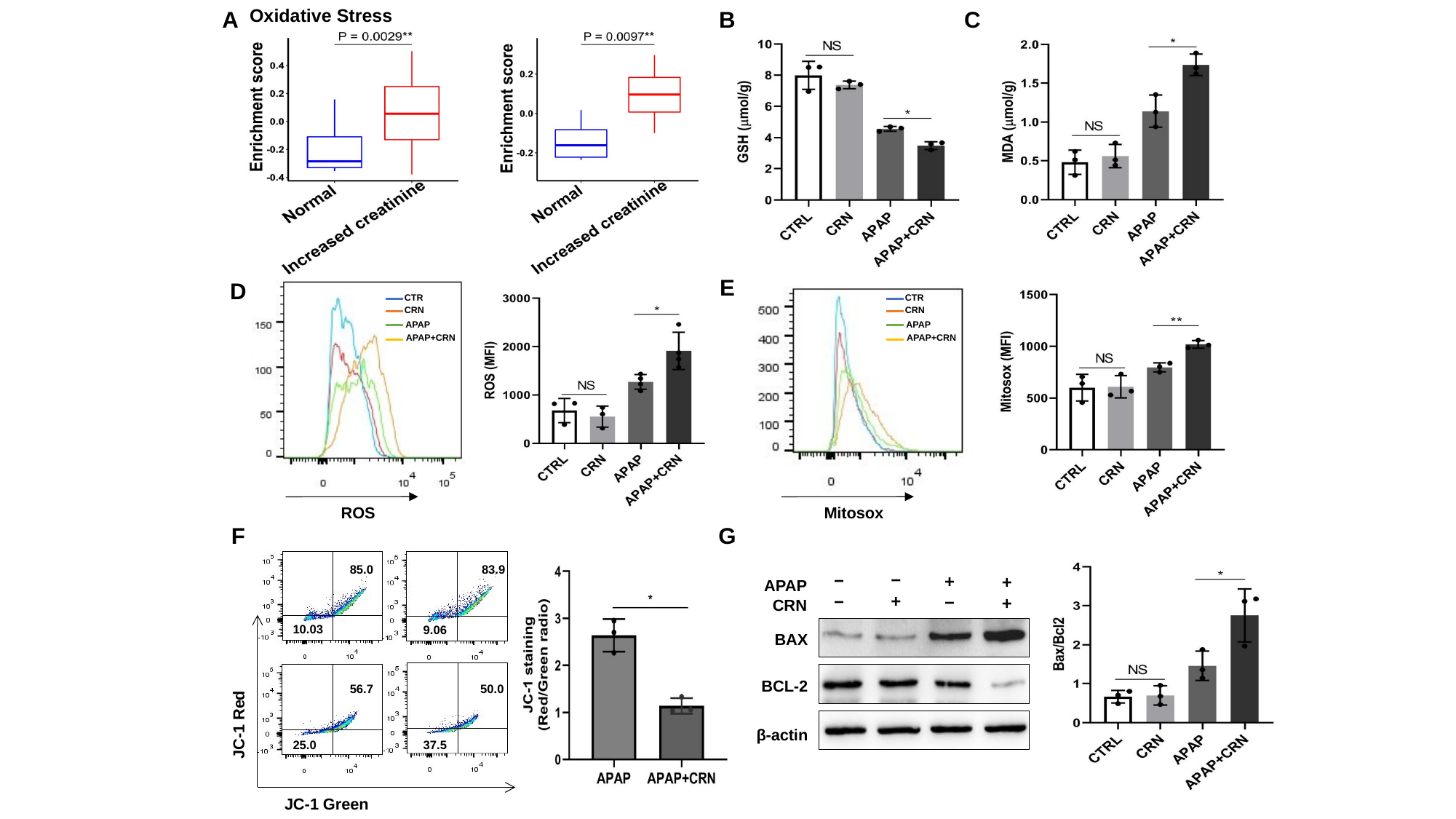

Oxidative Stress
A
B
C
E
D
CTR
CRN
APAP
APAP+CRN
CTR
CRN
APAP
APAP+CRN
ROS
Mitosox
F
G
85.0
83.9
− +
− −
+ −
++
APAPCRN
BAX
BCL-2
β-actin
10.03
9.06
56.7
50.0
JC-1 Red
25.0
37.5
JC-1 Green

## Slide 5
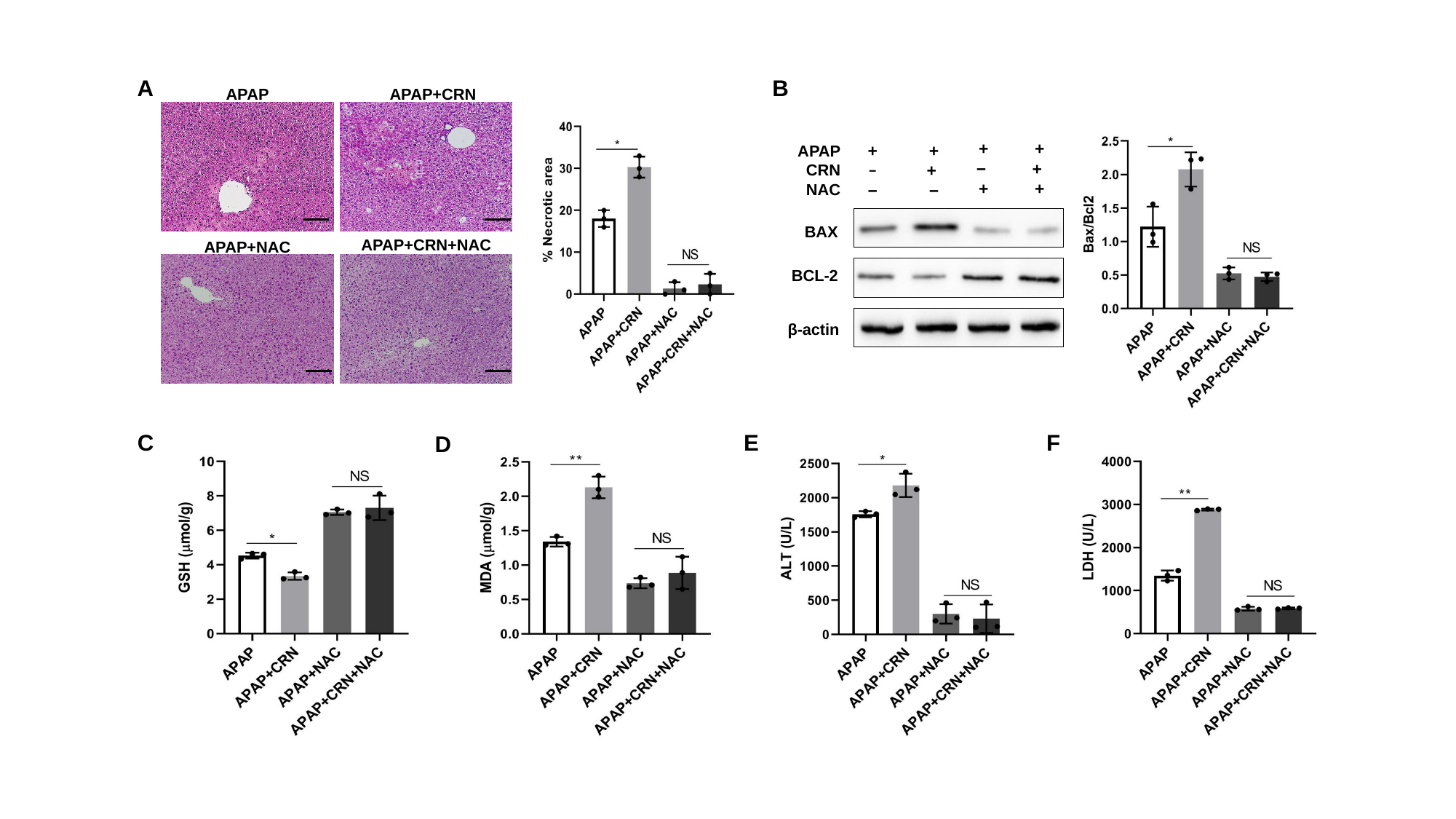

A
B
APAP
APAP+CRN
+
−
+
+
+
+
+
−
−
+
+
−
APAPCRN
NAC
BAX
APAP+CRN+NAC
APAP+NAC
BCL-2
β-actin
C
E
F
D

## Slide 6
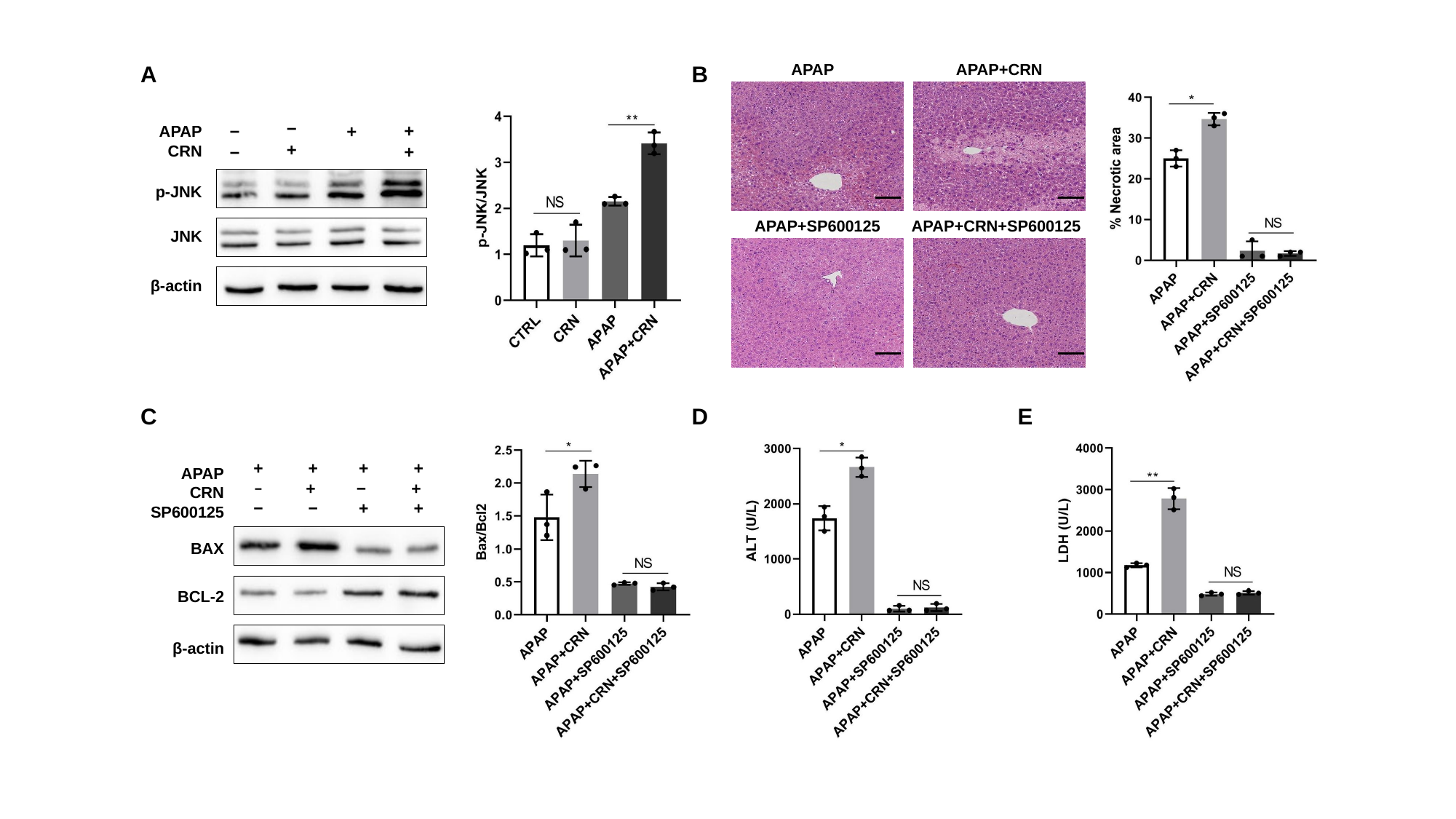

A
B
APAP
APAP+CRN
− +
++
− −
+ −
APAP
CRN
p-JNK
JNK
β-actin
APAP+SP600125
APAP+CRN+SP600125
C
D
E
+
−
−
+
+
−
+
−
+
+
+
+
APAP
CRN
SP600125
BAX
BCL-2
β-actin

## Slide 7
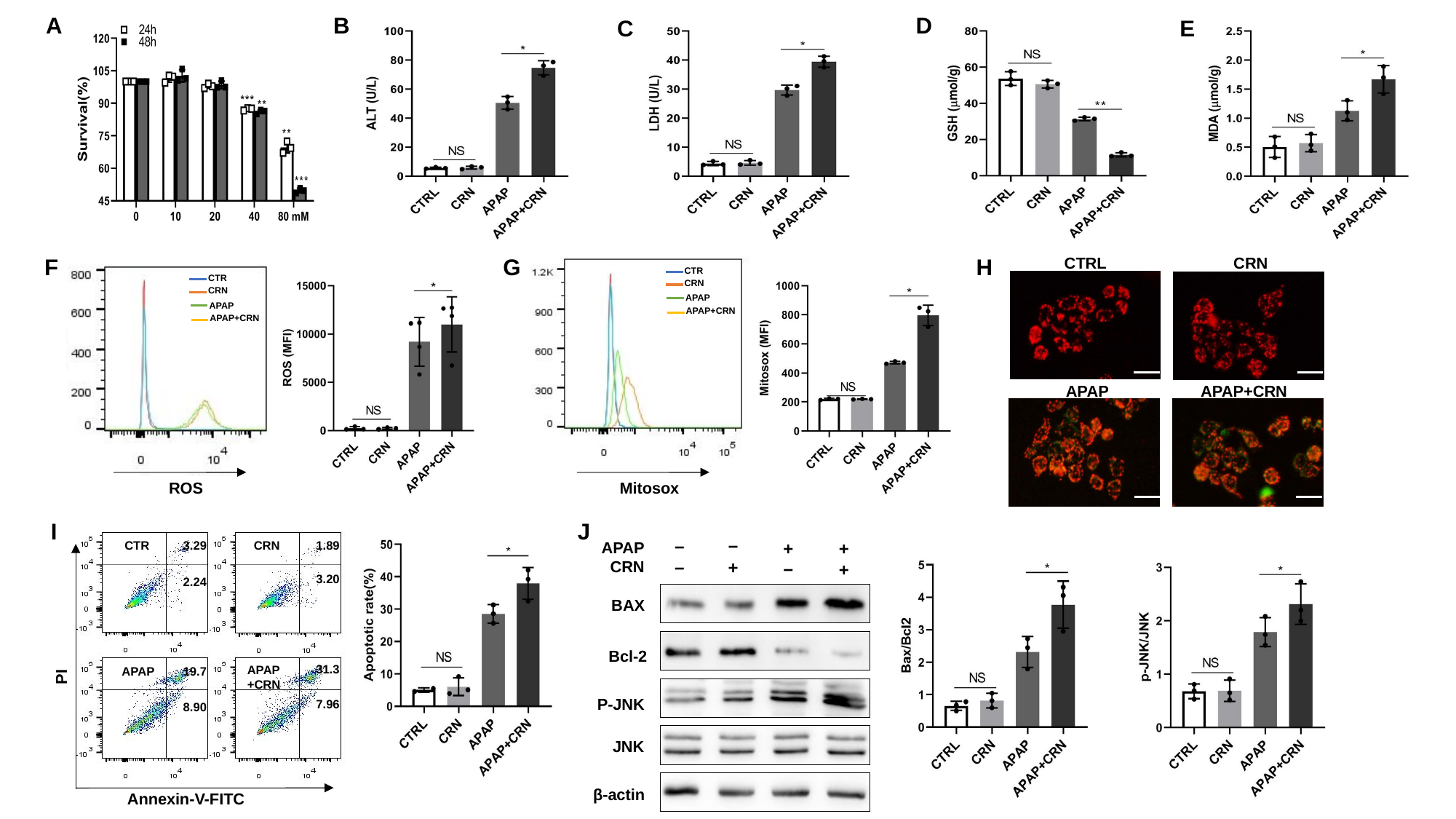

A
B
D
C
E
F
G
H
CTRL
CRN
APAP
APAP+CRN
CTR
CRN
APAP
APAP+CRN
CTR
CRN
APAP
APAP+CRN
ROS
Mitosox
CTR
CRN
3.29
1.89
PI
Annexin-V-FITC
3.20
2.24
31.3
APAP+CRN
19.7
 APAP
7.96
8.90
I
J
− +
− −
 APAP
CRN
+ −
++
BAX
Bcl-2
P-JNK
JNK
β-actin
