# Supplementary material for: Creatinine accelerates APAP-induced liver damage by increasing oxidative stress through ROS/JNK signaling pathway
Source: Front Pharmacol. 2022 Aug 24;13:959497. doi: 10.3389/fphar.2022.959497 (PMC9449354; doi:10.3389/fphar.2022.959497)
Supplement: Supplementary file 7 [file DataSheet1.doc]

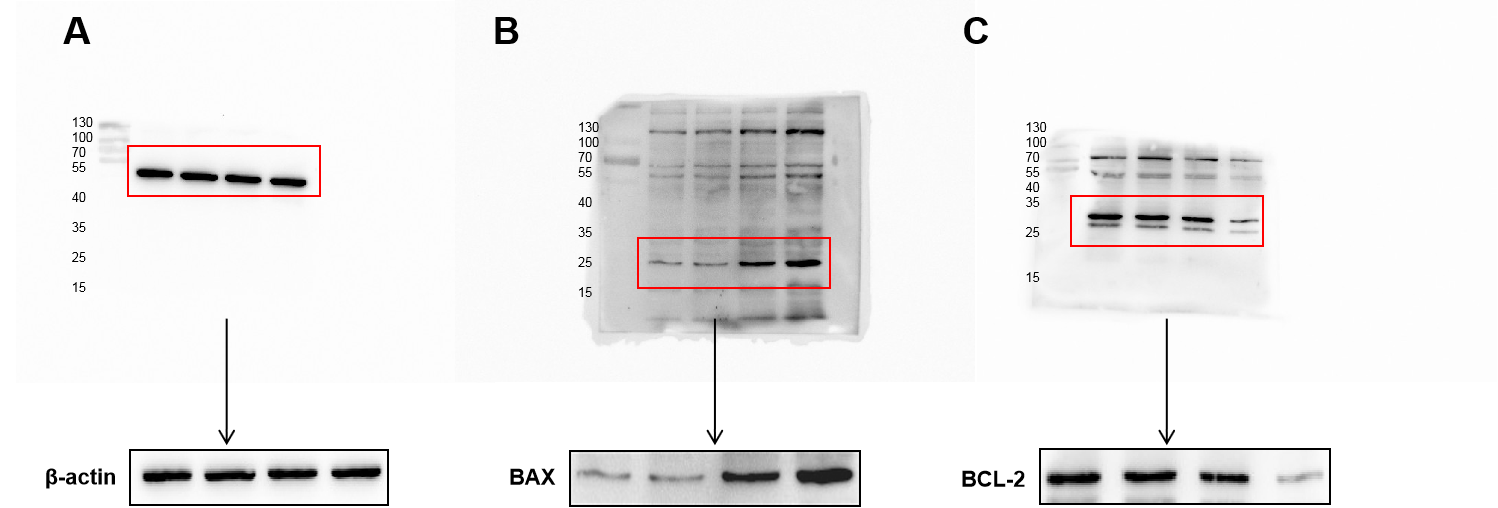


**Supplemental Fig.1 The full uncropped and unedited versions of the Western blots.**

From (A) to (C), the uncropped strip of western blots including β-actin, Bax and BCL2 corresponding to Fig.4G were showed and the edited version in red frame were used in article.


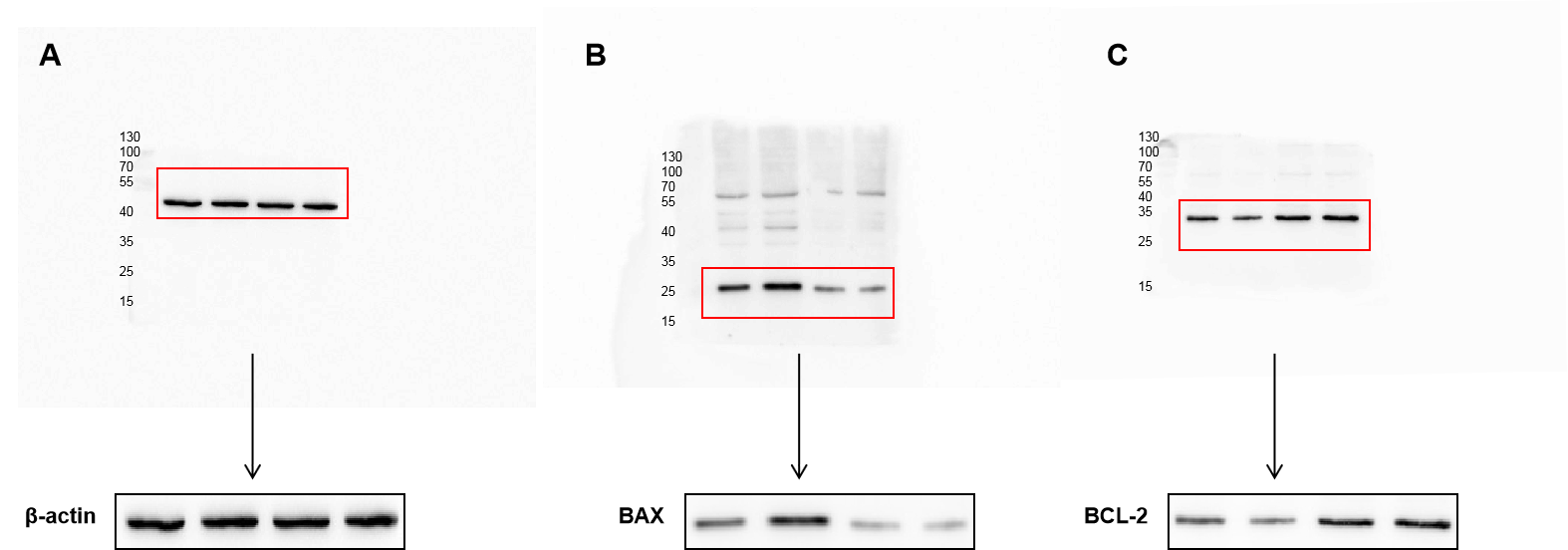


**Supplemental Fig.2 The uncropped and unedited versions of the Western blots.**

(A)-(C), the uncropped strip and edited version including β-actin, Bax and BCL2 were displayed which were corresponded to Fig.5B.


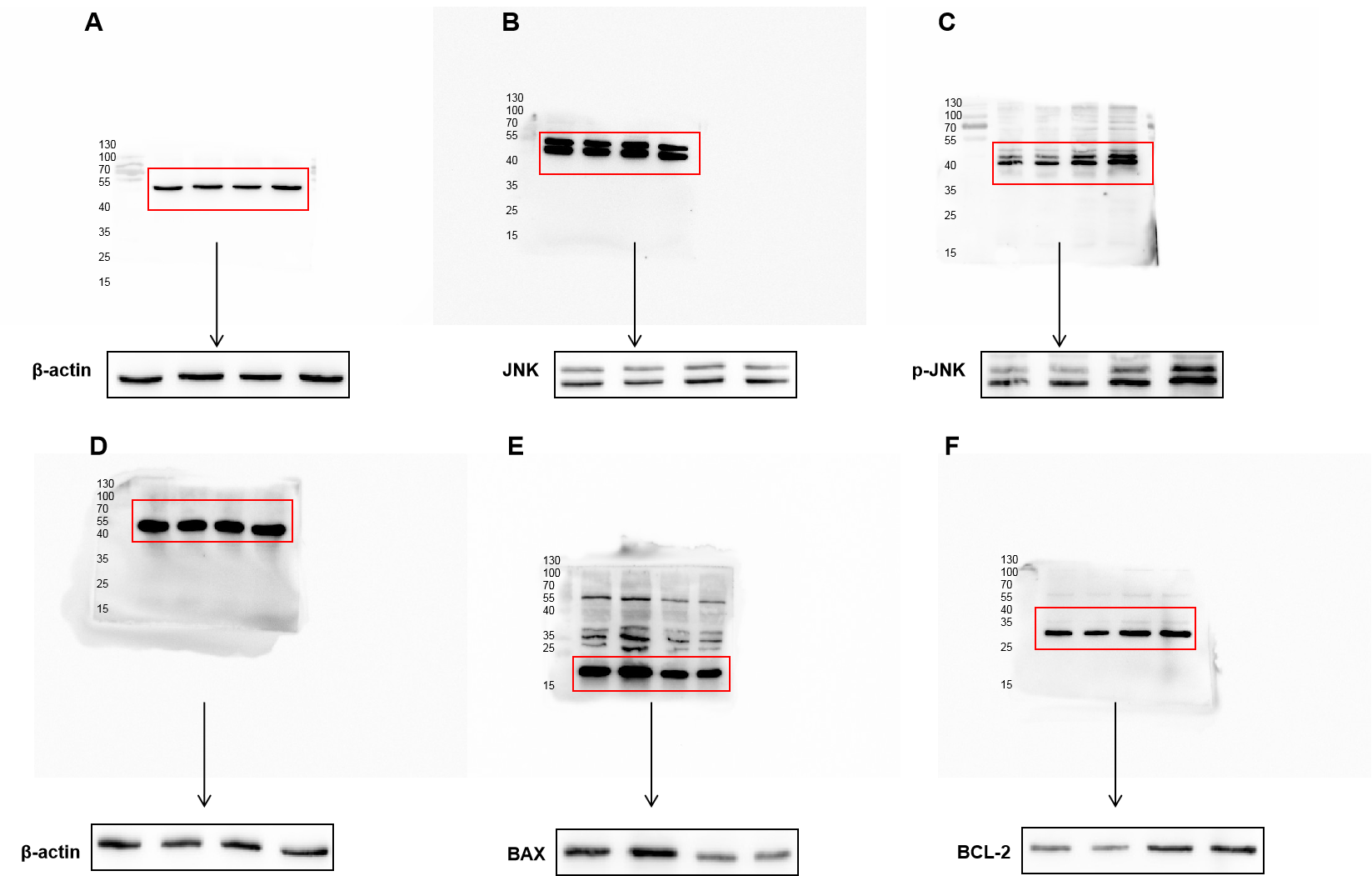


**Supplemental Fig.3 The uncropped and unedited pictures of the Western blots corresponding to Fig.6A and Fig.6C.** The uncropped strip and edited version of β-actin, JNK and p-JNK which corresponded to Fig.6A were showed from (A)-(C). The unedited and processed strips of β-actin, Bax and BCL2 in Fig.6B in the paper were showed from (D) and (F).


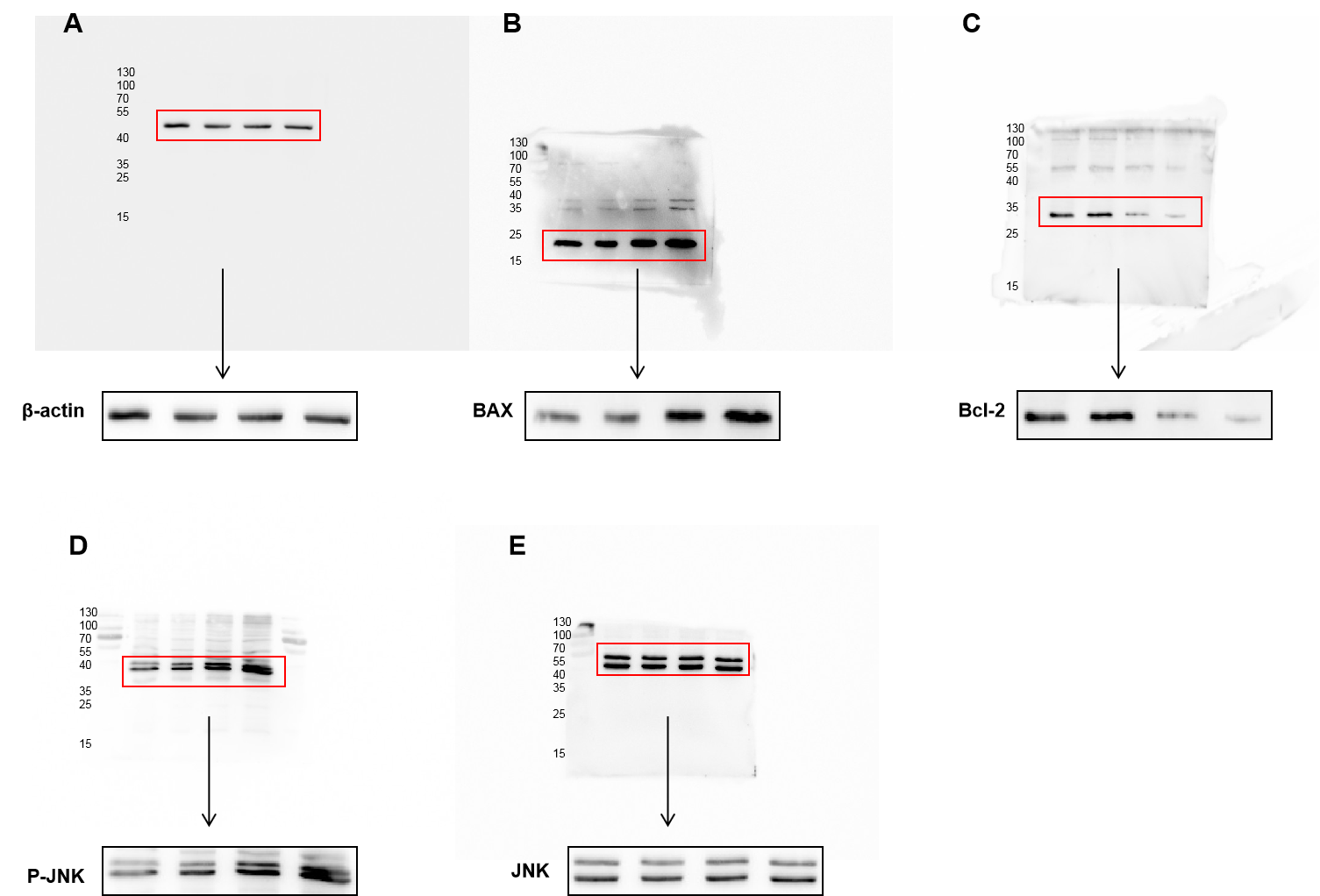


**Supplemental Fig.4 The uncropped and unedited pictures of the Western blots corresponding to Fig.7J.** The uncropped strip and edited version of β-actin, Bax, BCL2, JNK and p-JNK which were corresponded to Fig.7J were showed from (A)-(E).


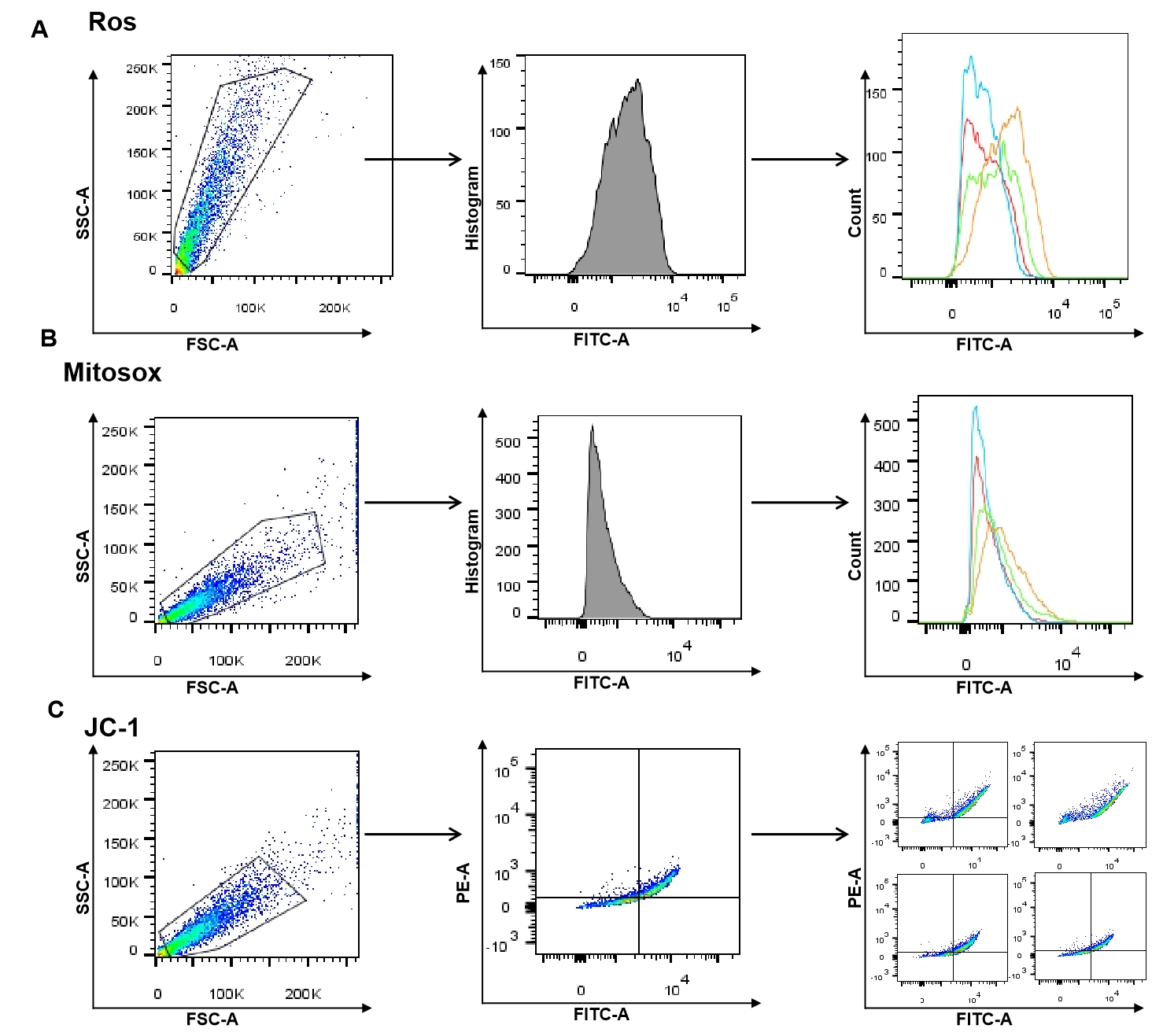


**Supplemental Fig.5 The flow strategies for Ros, Mitosox and JC-1.**

(A) The flow strategy of Ros which corresponded to Fig.3D was displayed. (B) The flow gate strategy for Mitosox corresponding to Fig.3E was showed. (C) The flow management strategy for JC-1corresponding to Fig.3F was demonstrated.


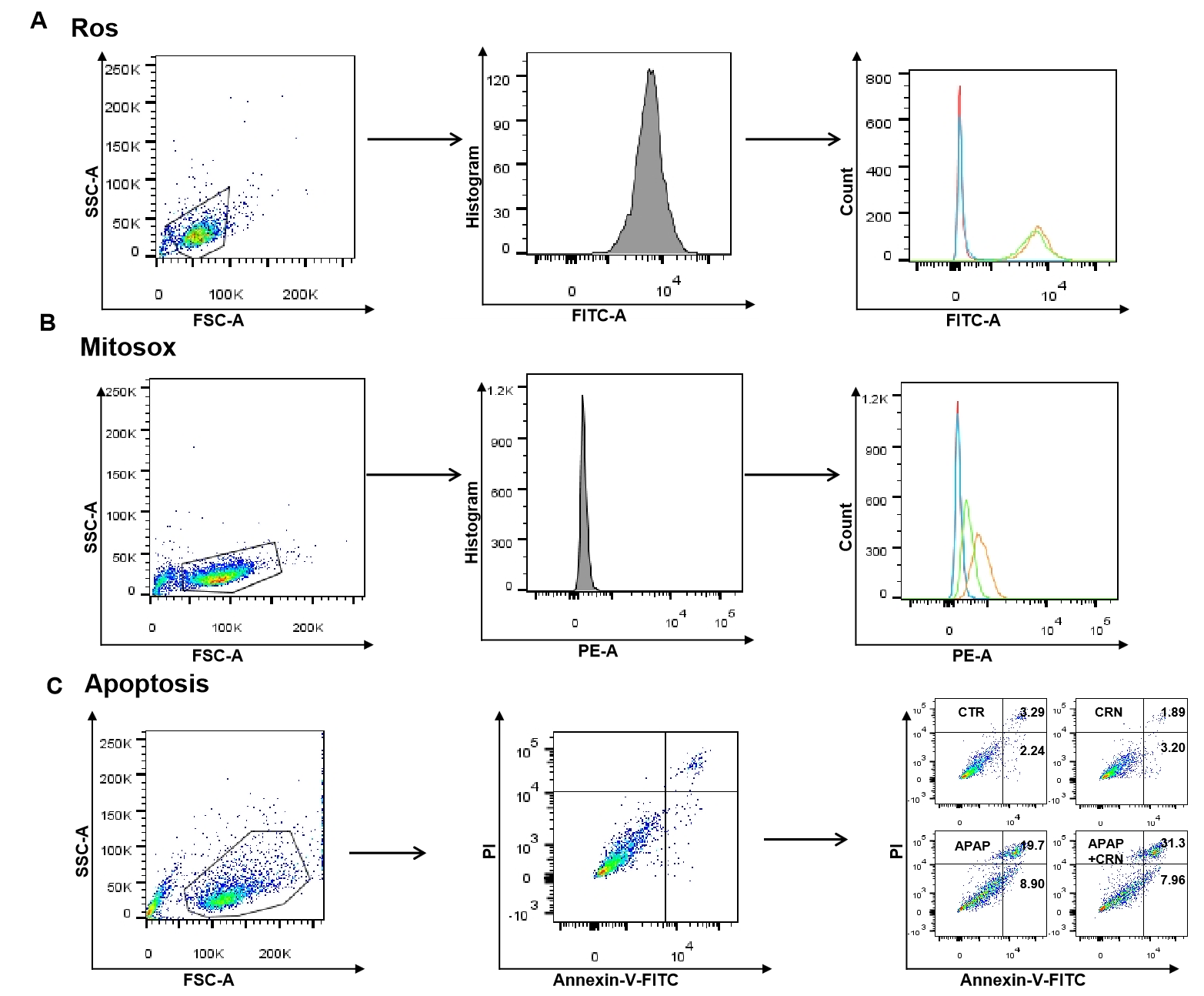


**Supplemental Fig.6 The flow schemes for Ros, Mitosox and apoptosis.**

(A) The flow strategy of Ros which was corresponded to Fig.7F was showed. (B) It was the flow gate strategy for Mitosox corresponding to Fig.7G. (C) The flow management strategy about apoptosis corresponding to Fig.7I was illustrated.


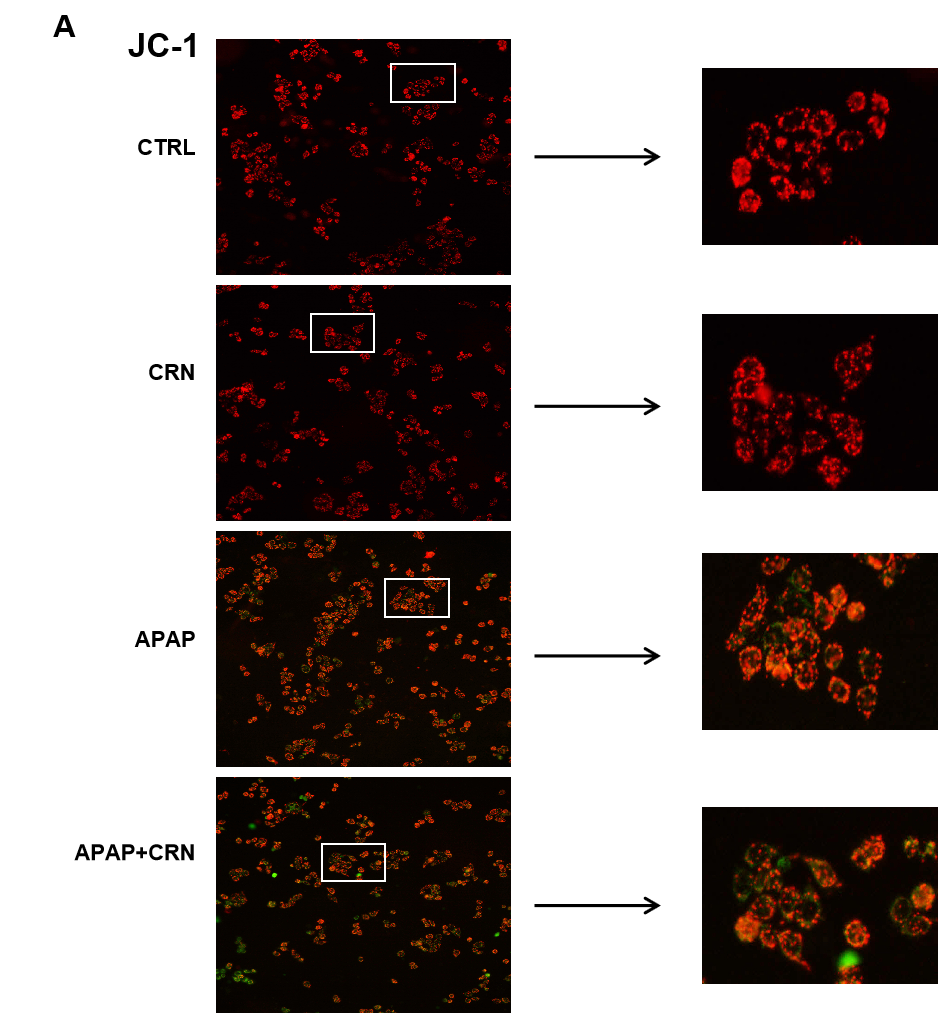


**Supplemental Fig.7 Mitochondrial membrane potential detection.**

The Mitochondrial membrane potential was detected by JC-1 staining using Fluorescence microscopy and the cells shown in the Fig.7H were circled.
